# The complete mitochondrial genome of the Caribbean spiny lobster *Panulirus argus*

**DOI:** 10.1038/s41598-018-36132-6

**Published:** 2018-12-06

**Authors:** J. Antonio Baeza

**Affiliations:** 1 Department of Biological Sciences, 132 Long Hall, Clemson University, Clemson, SC 29634 USA; 2 Smithsonian Marine Station at Fort Pierce, 701 Seaway Drive, Fort Pierce, Florida 34949 USA; 3 Departamento de Biología Marina, Facultad de Ciencias del Mar, Universidad Católica del Norte, Larrondo 1281, Coquimbo, Chile

## Abstract

*Panulirus argus* is a keystone species and target of the most lucrative fishery in the Caribbean region. This study reports, for the first time, the complete mitochondrial genome of *Panulirus argus* (average coverage depth nucleotide^−1^ = 70×) assembled from short Illumina 150 bp PE reads. The AT-rich mitochondrial genome of *Panulirus* *argus* was 15 739 bp in length and comprised 13 protein-coding genes (PCGs), 2 ribosomal RNA genes, and 22 transfer RNA genes. A single 801 bp long intergenic space was assumed to be the D-loop. Most of the PCGs were encoded on the H-strand. The gene order observed in the mitochondrial genome of *Panulirus argus* corresponds to the presumed Pancrustacean ground pattern. K_A_/K_S_ ratios calculated for all mitochondrial PCGs showed values < 1, indicating that all these PCGs are evolving under purifying selection. A maximum likelihood phylogenetic analysis (concatenated PCGs [n = 13], 154 arthropods) supported the monophyly of the Achelata and other infraorders within the Decapoda. Mitochondrial PCGs have enough phylogenetic informativeness to explore high-level genealogical relationships in the Pancrustacea. The complete mitochondrial genome of the Caribbean spiny lobster *Panulirus* *argus* will contribute to the better understanding of meta-population connectivity in this keystone overexploited species.

## Introduction

Within the order Decapoda, one of the most species-rich and diverse crustacean clades^1^, spiny and slipper lobsters (infraorder Achelata) exhibit a remarkable morphological, ecological, and behavioral disparity^2^. Recent studies on the Achelata have revealed remarkable traits and the conditions favoring their evolution. Examples include, among others, ontogenetic shifts in coloration, color pattern, and resource allocation to body parts (i.e., antenna, abdomen, tail fan) driven by decreasing predation risk with increasing body size^3^, active parental care in concert with large reproductive expenditure at large body sizes^4^, and the evolution of ‘behavioral immunity’ driven by viral pathogens^5^. Our knowledge of the biology of spiny lobsters has increased substantially over the past decades. Nonetheless, the ecology of numerous species remains unknown. Unfortunately, genomic resources are lacking in the infraorder Achelata and this lack of knowledge is limiting our understanding of morphological, ecological, and behavioral innovations in spiny and slipper lobsters. This study focuses on the development of genomic resources that are pivotal to improve our understanding of evolutionary innovations in this and other groups of crustaceans.

Within the Achelata, the Caribbean spiny lobster *Panulirus argus* (Latreille, 1804) is a keystone species in shallow water coral reefs^6^ and target of the most lucrative fishery in the greater Caribbean region^2^. The early life history of *P*. *argus* is well known[^7^ and references therein]. Adult females can produce 2–4 clutches of eggs per year with larger, older females reproducing earlier and having more clutches per year^8^. Fecundity ranges between 100,000 and 750,000 eggs per female and increases with female body size^4^. After completion of embryo development and hatching of larvae, 10 consecutive planktonic stages succeed one another^9^. These planktotrophic ‘phyllosomata’ larvae can spend 4–18 mo suspended in the water column^9^. The 10th larval stage undergoes a metamorphosis offshore, turning into a fast-swimming, lecithotrophic, short-lived (2–4 wks) ‘puerulus’ post-larval stage with morphology similar to that of juvenile and adult benthic lobsters, but almost devoid of coloration^10^. Pueruli actively swim from the open ocean to shallow coastal habitats, where they settle in vegetated habitats attracted by a set of cues, including metabolites of the red macroalgae *Laurencia* spp. and conspecifics^7^. Feeding resumes immediately after molting to the first fully benthic juvenile stage^11^. Juvenile and subadult lobsters are often found sharing crevice shelters^12^. The ecology of adult lobsters is less well understood. Perhaps more importantly, despite the commercial value and ecological importance of *P*. *argus*, few genomic resources exist for this species that could improve our understanding of its life cycle and the health of its populations^13,14^.

In this study, the complete sequence of the mitochondrial genome of *P*. *argus* is described. Nucleotide composition and codon usage profiles of protein coding genes (PCGs) were analyzed. The secondary structure of each identified tRNA gene was described and the putative D-loop/control region (CR) was examined in more detail. Selective constraints in PCGs, including those commonly used for population genetic inference, were explored. Lastly, the phylogenetic position of *P*. *argus* among other species of spiny lobsters (Decapoda: Achelata) and of the Achelata within the Decapoda was investigated based on mitochondrial PCGs.

## Methods

### Field collection and sequencing

Field collection was approved by FWCC (permit number: SAL-11-1319-SR).

One female of *P*. *argus* was collected in July 2017 by hand from a patch reef on the ocean side of Long Key (N24°49′26″; W80°48′48″), Florida, USA and transported alive to Clemson University, Clemson, SC. In the laboratory, the specimen was maintained in a 500 L circular polyethylene container. Muscle was extracted from a pereopod, and the tissue was immediately snap-frozen within a 50 ml centrifuge tube located inside a 3 L plastic ice chest containing dry ice blocks (−78.5 °C). Within an hour of tissue extraction, the sample was transported to OMEGA Bioservices (Norcross, GA, USA).

Total genomic DNA was extracted from the muscle tissue using the OMEGA BIO-TEK® E.Z.N.A.® Blood and Tissue DNA Kit following the manufacturer’s protocol. DNA concentration was measured using the QuantiFluor dsDNA system on a Quantus Fluorometer (Promega, Madison, WI, USA). A Kapa Biosystems HyperPrep kit (Kapa Biosystems, Wilmington, MA, USA) was used for whole genome library construction. Briefly, 1 µg of genomic DNA was fragmented using a Bioruptor sonicator (Diagenode, Denville, NJ, USA). DNA fragment ends were repaired, 3′ adenylated, and ligated to Illumina adapters. The resulting adapter-ligated libraries were PCR-amplified, Illumina indexes added, and pooled for multiplexed sequencing on an Illumina HiSeq X10 sequencer (Illumina, San Diego, CA, USA) using a pair-end 150 bp run format.

A total of 1.3071 billion reads were generated and made available in FASTQ format by Omega Bioservices. However, only 215 million reads were used for the mitochondrial genome assembly of *P*. *argus*.

### Mitochondrial genome assembly of *Panulirus argus*

Contaminants, low quality sequences (Phred scores < 30), Illumina adapters, and sequences with less than 50 bp were removed using the software Trimmomatic^15^, leaving 180 million (PE) high quality reads for the final mitogenome assembly. The mitogenome was built de novo using the NOVOPlasty pipeline v. 1.2.3^16^. NOVOPlasty uses a seed-and-extend algorithm that assembles organelle genomes from whole genome sequencing (WGS) data, starting from a related or distant single ‘seed’ sequence and an optional ‘bait’ reference mitochondrial genome^16^. To test the reliability of the assembly, I run NOVOPlasty using two strategies. First, I used a single fragment of the COI gene available in genebank (GU476034) as a seed. Second, I used the complete mitochondrial genome of *P*. *japonicus* (NC_004251) as a bait reference mitogenome in addition to the same partial COI seed. I chose to use the mitochondrial genome of *P*. *japonicus* as a ‘bait’ reference because it is the closely related congeneric species with a published mitochondrial genome available in Genebank^17^. The two runs used a kmer size of 49 following the developer’s suggestions^16^.

### Annotation and analysis of the *Panulirus argus* mitochondrial genome

The newly assembled mitochondrial genome was first annotated in the MITOS web server (http://mitos.bioinf.uni-leipzig.de)^18^ using the invertebrate genetic code. Annotation curation and start + stop codons corrections were performed using MEGA6^19^ and Expasy (https://web.expasy.org/). Genome visualization was conducted with OrganellarGenomeDRAW (http://ogdraw.mpimp-golm.mpg.de/index.shtml)^20^. The open reading frames (ORFs) and codon usage profiles of PCGs were analyzed. Codon usage for each PCG was predicted using the invertebrate mitochondrial code in the Codon Usage web server (http://www.bioinformatics.org/sms2/codon_usage.html). tRNA genes were identified in the software ARWEN^21^ as implemented in the MITOS web server and the secondary structure of each tRNA was predicted using the tRNAscan-SE v.2.0 web server (http://trna.ucsc.edu/tRNAscan-SE/)^22^. tRNA secondary structures were visualized in the RNAfold web server (http://rna.tbi.univie.ac.at/cgi-bin/RNAWebSuite/RNAfold.cgi)^23^.

The putative D-loop/CR of *P*. *argus* was examined in more detail. The number of repeats in the region was investigated with the Tandem Repeat Finder Version 4.09 web server (http://tandem.bu.edu/trf/trf.html)^24^. DNA motifs were discovered in the putative D-loop/CR of *P*. *argus* using the default options in MEME^25^. I also aligned the putative D-loop/CR of *P*. *argus* with that of four congeneric species (*P*. *cygnus* [KT696496], *P*. *japonicus* [AB071201], *P*. *stimpsoni* [GQ292768], and *P*. *versicolor* [KC107808]) and used the GLAM2 algorithm^26^ to discover short motifs (<100 bp) containing gaps. Mfold (http://unafold.rna.albany.edu/) and Quickfold (http://unafold.rna.albany.edu/?q=DINAMelt/Quickfold) web servers were used to predict the secondary structure of this region with particular attention to the presence of stem-loops towards the end of the sequence.

Selective constraints in PCGs, including those commonly used for population genetic inference in decapod crustaceans and other marine invertebrates (i.e., Cox1, CytB), were explored. Overall values of K_A_ (the number of nonsynonymous substitutions per nonsynonymous site: K_A_ = d_N_ = S_A_/L_A_), K_S_ (number of synonymous substitutions per synonymous site: K_S_ = d_S_ = S_S_/L_S_), and ω (the ratio K_A_/K_S_) were estimated for each PCG in the software KaKs_calculator 2.0^27^. The above values were based on a pairwise comparison between *P*. *argus* and the closely related *P*. *japonicus*. Next, to identify positively selected sites along the length of each examined sequence, the values of K_A_, Ks, and ω were also calculated while adopting a sliding window (window length = 52, step length = 12) that ‘slipped’ along each sequence. The γ-MYN model^28^ was used during calculations to account for variable mutation rates across sequence sites^27^. If PCGs are under no selection, positive selective constraint (purifying selection), or diversifying selection, the ratio ω (=KA/KS) is expected to be equal to 1, >1, or <1, respectively^27^.

The phylogenetic position of *P*. *argus* among other species of spiny lobsters (Decapoda: Achelata) was examined. The newly assembled and annotated mitochondrial genome of *P*. *argus* and those of a total of 153 other species of arthropods, including members of the Achelata, available in the Genebank database were used for the phylogenetic analysis conducted using the MitoPhAST pipeline^29^. The phylogenetic analysis included a total 154 terminals belonging to 146 different genera, and representatives of 14 infraorders, orders, or superfamilies in the subphylum Crustacea, class Malacostraca. The full list of species used for phylogenetic analysis is available in Supplementary Table S1. MitoPhAST extracts all 13 PCG nucleotide sequences from species available in Genbank and others provided by the user (i.e., *P*. *argus*), translates each PCG nucleotide sequence to amino acids, conducts alignments for each PCG amino acid sequence using Clustal Omega^30^, removes poorly aligned regions with trimAl^31^, partitions the dataset and selects best fitting models of sequence evolution for each PCG with ProtTest^32^, and uses the concatenated and partitioned PCG amino acid alignment to perform a maximum likelihood phylogenetic analysis in the software RaxML^33^. The full matrix of species by genes used for phylogenetic analysis is available in Supplementary Table S2. The robustness of the ML tree topology was assessed by bootstrap iterations of the observed data 1,000 times.

## Results and Discussion

**Figure 1:**
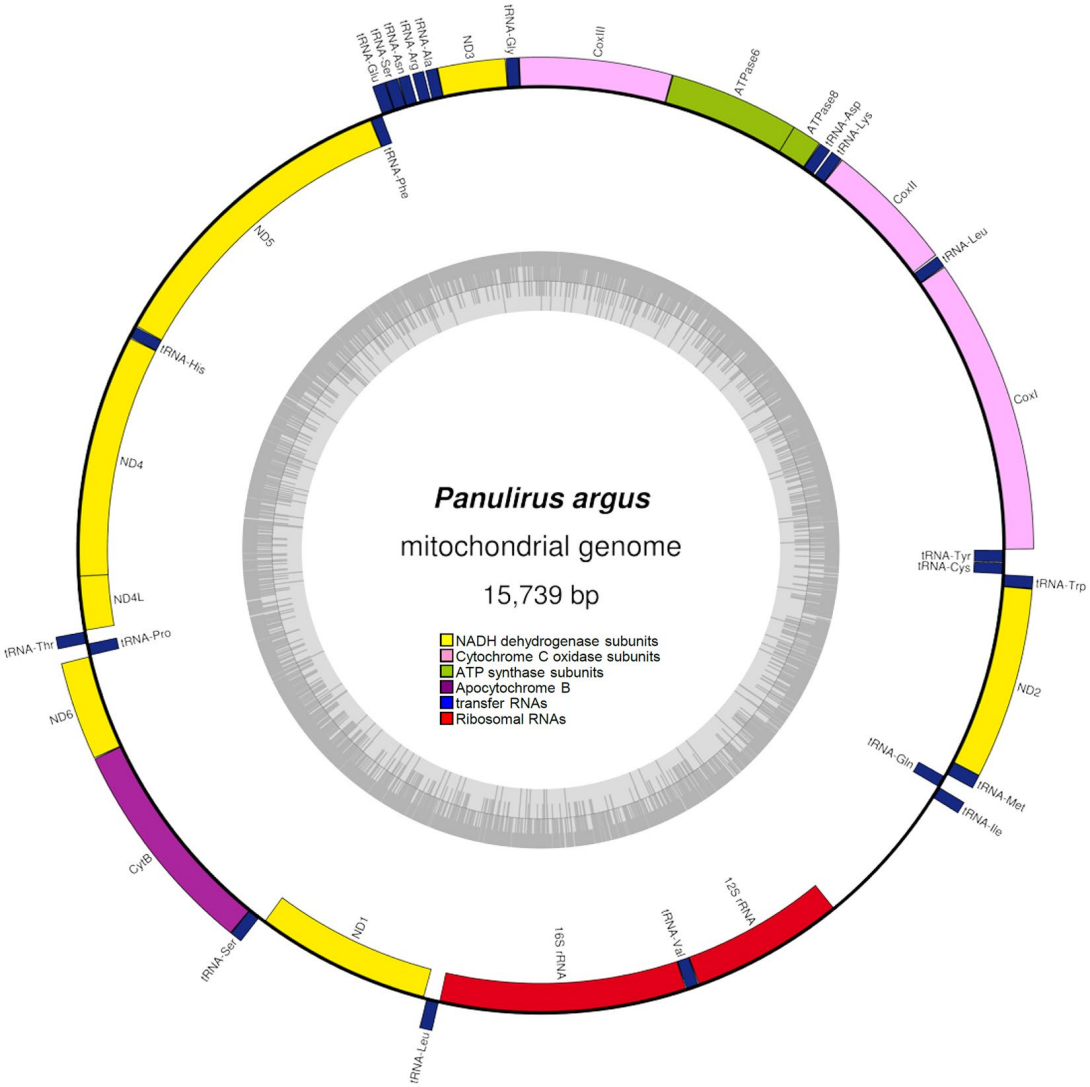
Circular genome map of *Panulirus argus* mitochondrial DNA. The map is annotated and depicts 13 protein-coding genes (PCGs), 2 ribosomal RNA genes (rrnS [12S ribosomal RNA] and rrnL [16S ribosomal RNA]), 22 transfer RNA (tRNA) genes, and the putative control region. The inner circle depicts GC content along the genome. The putative D-Loop/Control region is not annotated.

The two strategies employed to assemble the mitochondrial genome of *Panulirus argus* in NOVOPlasty resulted in identical sequences. The complete mitochondrial genome of *P*. *argus* (GeneBank accession number MH068821) was 15739 bp in length and comprised 13 protein-coding genes (PCGs), 2 ribosomal RNA genes (rrnS [12S ribosomal RNA] and rrnL [16S ribosomal RNA]), and 22 transfer RNA (tRNA) genes. Most of the PCGs and tRNA genes were encoded on the H-strand. Only 4 PCGs (nad5, nad4, nad4l, and nad1) and 8 tRNA genes (trnF, trnH, trnP, trnL1, trnV, trnQ, trnC, trnY) were encoded in the L-strand. The 2 ribosomal RNA genes were encoded in the L-strand (Fig. 1, Table 1). The gene order observed in *P*. *argus* is identical to that reported before in the genus *Panulirus*^34–39^ and also corresponds to the presumed Pancrustacean (Hexapoda + Crustacea) ground pattern^40^. Gene overlaps comprising a total of 24 bp were observed in 9 gene junctions: atp8-atp6 (overlap = 7 bp), atp6-cox3 (1 bp), cox3-trnG (1 bp), nad3-trnA (2 bp), trnS1-trnE (1 pb), nad4-nad4l (7 pb), cob-trnS2 (1 pb), trnI-trnQ (3 pb), and trnW-trnY (1 pb) (Fig. 1, Table 1). In turn, short intergenic spaces ranging in size between 1 and 32 bp were observed in a total of 11 gene junctions. A single long intergenic space involving 801 bp in the mitochondrial genome of *P*. *argus* was assumed to be the D-loop/CR (Fig. 1, Table 1).

**Table 1:** Mitochondrial genome of *Panulirus argus*. Arrangement and annotation.

Name	Type	Start	Stop	Strand	Length (bp)	Start	Stop	Anticodon	Inter Genic space
Cox1	Coding	1	1534	+	1534	ACG	T		0
trnL2(tta)	tRNA	1535	1598	+	64			TAA	4
cox2	Coding	1603	2290	+	688	ATG	T		0
trnK(aaa)	tRNA	2291	2355	+	65			TTT	7
trnD(gac)	tRNA	2363	2426	+	64			GTC	0
atp8	Coding	2427	2585	+	159	ATG	TAA		0
atp6	Coding	2579	3256	+	678	ATG	TAA		−7
cox3	Coding	3256	4047	+	792	ATG	TAA		−1
trnG(gga)	tRNA	4047	4113	+	67			TCC	−1
nad3	Coding	4114	4467	+	354	ATC	TAG		0
trnA(gca)	tRNA	4466	4529	+	64			TGC	−2, 5
trnR(cga)	tRNA	4535	4598	+	64			TCG	11
trnN(aac)	tRNA	4610	4673	+	64			GTT	0
trnS1(aga)	tRNA	4674	4741	+	68			TCT	0
trnE(gaa)	tRNA	4741	4811	+	71			TTC	−1
trnF(ttc)	tRNA	4812	4878	—	67			GAA	1
nad5	Coding	4879	6607	—	1729	ATG	T		0
trnH(cac)	tRNA	6608	6674	—	67			GTG	0
nad4	Coding	6675	8013	—	1339	ATG	T		0
nad4l	Coding	8007	8309	—	303	ATG	TAA		−7, 2
trnT(aca)	tRNA	8312	8378	+	67			TGT	0
trnP(cca)	tRNA	8379	8446	—	68			TGG	2
nad6	Coding	8449	8964	+	516	ATC	TAA		0
cob	Coding	8965	10101	+	1137	ATG	TGA		0
trnS2(tca)	tRNA	10100	10167	+	68			TGA	−1, 32
nad1	Coding	10200	11165	—	966	ATG	TAG		13
trnL1(cta)	tRNA	11179	11248	—	70			TAG	0
rrnL	rRNA	11249	12605	—	1357	—	—		0
trnV(gta)	tRNA	12606	12676	—	71			TAC	0
rrnS	rRNA	12677	13524	—	848	—	—		0
CR ^Putative^		13525	14326	+	801				0
trnI(atc)	tRNA	14327	14394	+	68			GAT	0
trnQ(caa)	tRNA	14392	14460	—	69			TTG	−3, 13
trnM(atg)	tRNA	14474	14541	+	68			CAT	0
nad2	Coding	14542	15543	+	1002	ATG	TAA		0
trnW(tga)	tRNA	15542	15608	+	67			TCA	−2
trnC(tgc)	tRNA	15608	15671	—	64			GCA	−1, 1
trnY(tac)	tRNA	15673	15739	—	67			GTA	0

Twelve out of the 13 PCGs in the mitochondrial genome of *Panulirus argus* exhibited conventional invertebrate and arthropod/crustacean mitochondrial start codons (ATG, ATC) (Table 1). Cox1 exhibited an alternative putative start codon (CGA) as previously observed in other spiny lobsters and decapod crustaceans (^34,38,39,41^, and references therein). Nine PCGs ended with a complete and conventional termination codon. Six genes (atp8, atp6, cox3, nad4l, nad6 and nad2) ended with TAA, two genes ended with TAG (nad3 and nad1) and one gene (CytB) ended with TGA. Cox1, cox2, nad4 and nad5 terminated with an incomplete stop codon T, as often observed in other arthropod, including crustacean, mitochondrial genomes^34^. Truncated stop codons are hypothesized to be completed via post-transcriptional poly-adenylation^42^.

The mitochondrial genome of *Panulirus argus* contained an A + T bias with an overall base composition of A = 32.9%, T = 29.7%, C = 22.7%, and G = 14.6%. This A + T bias is within the known range reported for mitochondrial genomes in spiny lobsters and other decapod crustaceans and probably affects codon usage^34^. In the PCGs of *P*. *argus*, the most frequently used codons were UUU (Phe, N = 189 times used, 5.07% of the total), UUC (Phe, N = 131, 3.51%), AUU (Ile, N = 185, 4.96%), and UUA (Leu, N = 167, 4.48%). Less frequently used codons included CGG (Arg, N = 9, 0.24%), CGC (Arg, N = 8, 0.21%), and AGC (Ser, N = 8, 0.21%) (Table 2).

**Table 2:** Codon usage analysis of PCGs in the mitochondrial genome of *Panulirus argus*.

AA	Codon	N	/1000	Freq	AA	Codon	N	/1000	Freq
Ala	GCG	22	5.99	0.11	Pro	CCG	30	8.04	0.20
	GCA	54	14.47	0.27		CCA	36	9.65	0.24
	GCT	76	20.37	0.38		CCT	45	12.06	0.31
	GCC	49	13.13	0.24		CCC	36	9.65	0.24
Cys	TGT	31	8.31	0.63	Gln	CAG	26	6.97	0.38
	TGC	18	4.82	0.37		CAA	43	11.53	0.62
Asp	GAT	43	11.53	0.57	Arg	CGG	9	2.41	0.15
	GAC	32	8.58	0.43		CGA	31	8.31	0.51
Glu	GAG	31	8.31	0.35		CGT	13	3.48	0.21
	GAA	57	15.28	0.65		CGC	8	2.14	0.13
Phe	TTT	189	50.66	0.59	Ser	AGG	35	9.38	0.08
	TTC	131	35.11	0.41		AGA	49	13.13	0.12
Gly	GGG	67	17.96	0.27		AGT	39	10.45	0.09
	GGA	83	22.25	0.34		AGC	8	2.14	0.02
	GGT	61	16.35	0.25		TCG	43	11.53	0.10
	GGC	35	9.38	0.14		TCA	92	24.66	0.22
His	CAT	40	10.72	0.51		TCT	110	29.48	0.26
	CAC	38	10.18	0.49		TCC	47	12.60	0.11
Ile	ATT	185	49.58	0.61	Thr	ACG	29	7.77	0.15
	ATC	119	31.89	0.39		ACA	58	15.55	0.30
Lys	AAG	41	10.99	0.45		ACT	76	20.37	0.39
	AAA	51	13.67	0.55		ACC	32	8.58	0.16
Leu	TTG	101	27.07	0.18	Val	GTG	42	11.26	0.15
	TTA	167	44.76	0.29		GTA	79	21.17	0.29
	CTG	45	12.06	0.08		GTT	103	27.61	0.37
	CTA	112	30.02	0.20		GTC	52	13.94	0.19
	CTT	100	26.80	0.17	Trp	TGG	36	9.65	0.37
	CTC	48	12.87	0.08		TGA	61	16.35	0.63
Met	ATG	98	26.27	0.51	Tyr	TAT	74	19.83	0.57
	ATA	95	25.46	0.49		TAC	55	14.74	0.43
Asn	AAT	43	11.53	0.40	Stop	TAG	2	0.54	0.25
	AAC	64	17.15	0.60		TAA	6	1.61	0.75

**Figure 2:**
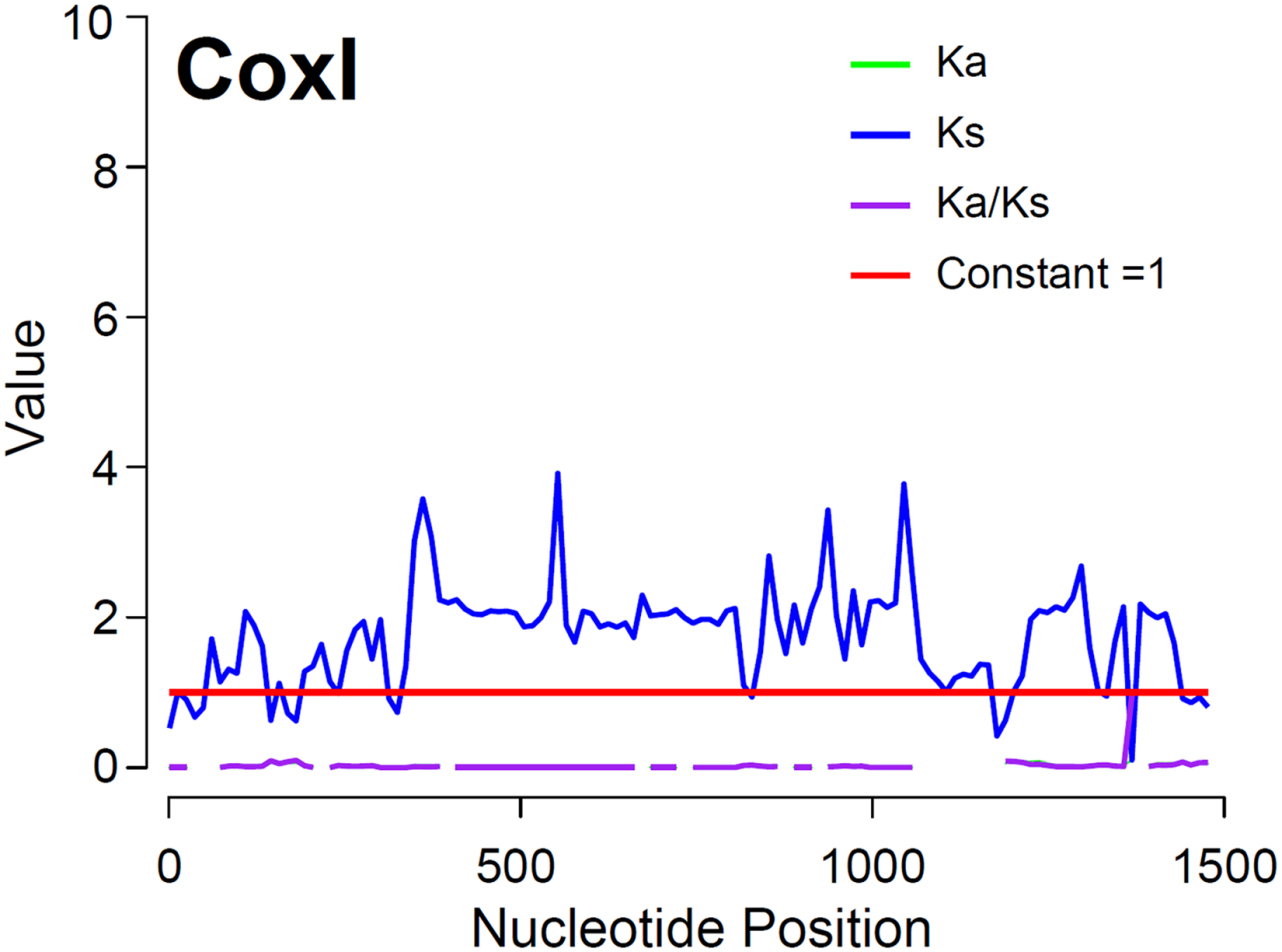
Selective pressure analysis in the Cox1 gene of *Panulirus argus*. K_A_, K_S_ and K_A_/K_S_ values were calculated using the γ-MYN model and adopting a sliding window of length = 52 and step length = 12. See methods and results for further details.

The K_A_/K_S_ ratios in all mitochondrial PCGs showed values < 1, indicating that all these PCGs are evolving under purifying selection. Examination of K_A_/K_S_ ratio values in sliding windows across the length of each PCG sequence further indicated that purifying selection is acting along the entire PCG sequence (Supplementary Fig. S1). Remarkably, the overall K_A_/K_S_ ratios observed for CytB and Cox1 (K_A_/K_S_ < 0.0035 and 0.0011, respectively) were an order of magnitude lower than those observed for the remaining PCGs (range: 0.011–0.081) suggesting strong purifying selection and evolutionary constraints in the former genes (Fig. 2). Selective pressure in mitochondrial PCGs has been poorly studied in decapod crustaceans but a similar pattern of widespread purifying selection in mitochondrial PCGs has been observed in other arthropods[^43^ and references therein].

**Figure 3:**
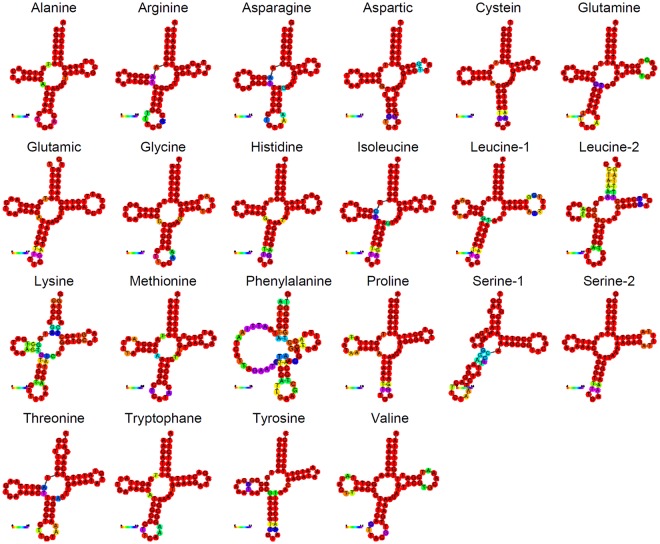
Secondary structure of tRNAs in the mitochondrial genome of *Panulirus argus* predicted by ARWEN and tRNAscan-SE v.2.0.

tRNA genes encoded in the mitochondrial genome of *P*. *argus* ranged in length from 64 to 71 bp and all but one exhibited a standard ‘cloverleaf’ secondary structure as predicted by both ARWEN and tRNAscan-SE v.2.0. Interestingly, the RNAfold web server was not able to enforce the secondary structure of the tRNA-F (Phenylalanine) gene predicted by ARWEN and tRNAscan-SE resulting in the reconstruction of a tRNA with the dihydrouridine (DHU) stem forming a simple loop (Fig. 3). In agreement to that reported for the closely related spiny lobster *P*. *japonicus* and other crustaceans [*Pagurus longicarpus*^40^; *Tigriopus japonicus*^34^], the tRNA-Lys and the tRNA-Ser1 genes in *P*. *argus* bear the anticodons TTT and TCT, respectively. By contrast, CTT and GCT are most often reported as anticodons for the tRNA-Lys and tRNA-Ser 1 genes in other invertebrate mitochondrial genomes^43^. The anticodon nucleotides of the remaining tRNA genes were identical to those found in other crustacean mitochondrial genomes^43^.

The rrnS and rrnL genes identified in the mitochondrial genome of *P*. *argus* were 848 and 1357 nucleotides long, respectively. The two genes were A + T biased. The overall base composition of the rrnL gene was A = 32.1%, T = 35.4%, C = 21.4%, and G = 11.1%, and that of the rrnS gene was A = 32.5%, T = 32.5%, C = 22.6%, and G = 12.3.6%. The rrnL gene is located between tRNA-L1 and tRNA-V. The rrnS gene is located close to the rrnL, between the tRNA-V gene and the relatively long non coding putative D-loop/CR (Fig. 1).

In *P*. *argus*, the 801 bp long intergenic region assumed to be the D-loop/CR is located between the 12 S ribosomal RNA and tRNA-I (Fig. 1). The region was A + T rich with an overall base composition: A = 37%, T = 32.6%, C = 20%, and G = 10.5%. Visual examination of the sequence and the Tamdem Repeat Finder web server analysis failed to detect tandemly repeated sequences in this region in disagreement to that observed in the Chinese spiny lobster *Panulirus stimpsoni*^35^ and other crustaceans (i.e., in the branchiopod genus *Daphnia*^44^). In some hexapod arthropods, the region is clearly divided into well defined motifs^43^; However, after aligning this 801 bp region in *P*. *argus* with that of 6 other species of *Panulirus* (*P*. *cygnus*, *P*. *versicolor*, *P*. *stimpsoni*, *P*. *homarus*, *P*. *ornatus*, and *P*. *japonicus*), GLAM2 recovered 2 AT-rich motifs. The first 35-pb long motif was located in the H-strand of the intergenic region (between 223–257 pb in the *P*. *argus* putative CR after alignment) while the second 31-pb long motif was located in the L-strand (727–757 pb). Secondary structure prediction analysis in Mfold and Quickfold (assuming 27 °C) resulted in seven and six possible folding configurations, respectively, with a change in Gibbs free energy (ΔG) ranging from −99.20 to −94.52 Kcal/mol (Supplementary Fig. S2). In Mfold as well as in Quickfold, four out of the 6–7 reconstructions featured stem-loop structures near the 3′ end of the region located between the bp 686 and 791 (Supplementary Fig. S2). A similar arrangement has been reported before in the putative mitochondrial genome control region of other invertebrates, including crustaceans^35,43,45^.

**Figure 4:**
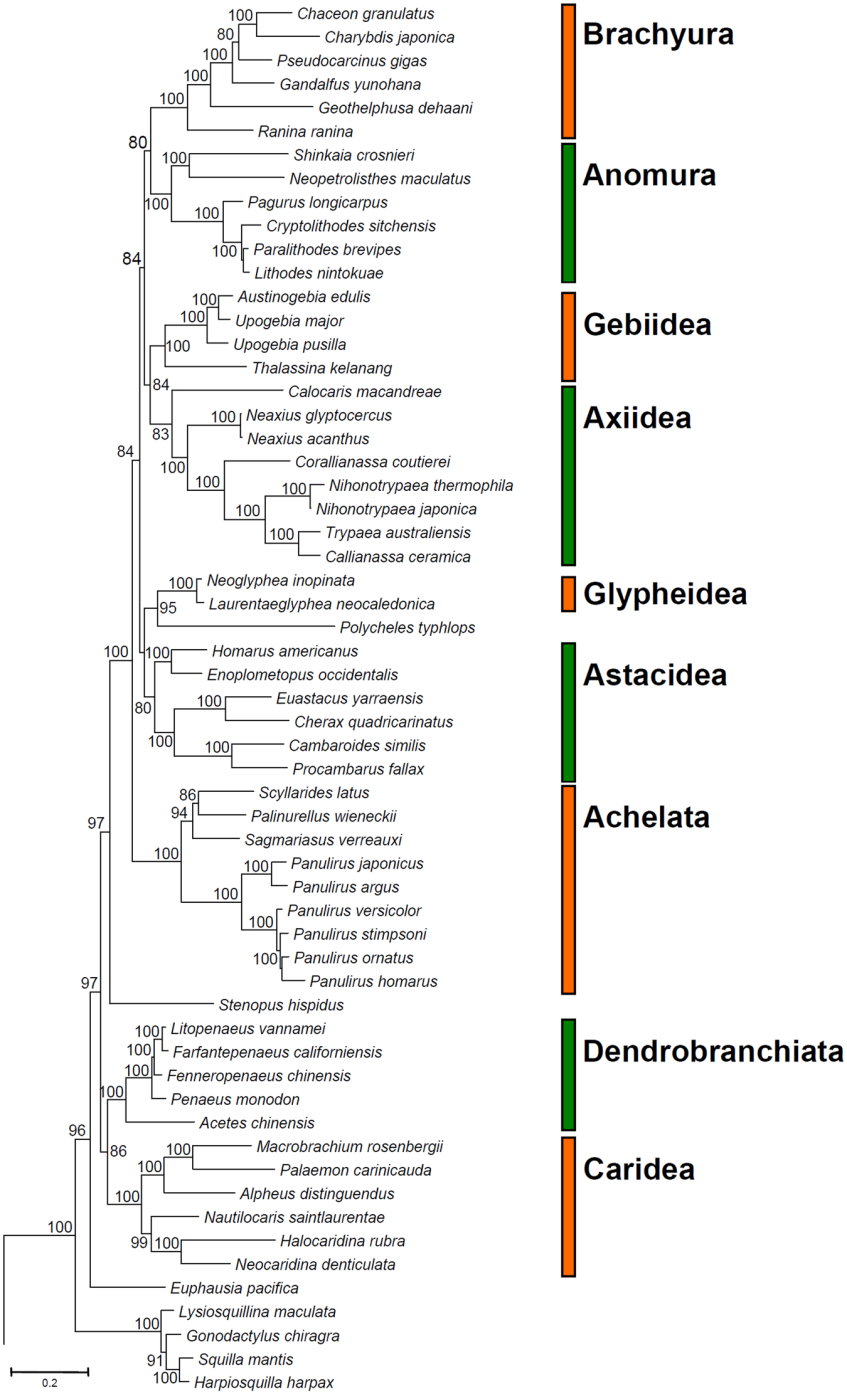
‘Total evidence’ phylogenetic tree obtained from ML analysis based on a concatenated alignment of amino acids of the 13 protein coding genes present in the mitochondrial genome of representatives of the subphylum Crustacea and allies. In the analysis, the horseshoe crab *Limulus polyphemus* (subphylum Chelicerata) was used as outgroup. Numbers above or below the branches represent bootstrap values. The analysis included a total of 154 species and 3144 amino acid characters. The optimal molecular evolution model found by ProtTest as implemented in NOVOPlasty was the mtZOA + F + I + G4 model that was applied to two different partitions (partition 1: ATP6 +ATP8 +NAD6 +NAD3 +NAD2 +COB +COX1 +COX2 +COX3, partition 2: NAD1 +NAD4 +NAD4L +NAD5) also found to be optimal for the dataset by ProtTest. For clarity, only the section of the tree containing species in the Decapoda is depicted. See Supplementary Fig. S3 online for full phylogenetic tree.

The ML phylogenetic tree (154 terminals, 3144 amino acid characters, 2451 informative sites) confirmed the monophyly of the Achelata and placed *P*. *argus* in a monophyletic clade with *P*. *japonicus*, in agreement with previous phylogenetic studies using a combination of partial mitochondrial and nuclear genes^46^ (Fig. 4 and Supplementary Fig. S3). Additional well supported clades within the Decapoda included the infraorders Brachyura, Anomura, Gebiidea, Glypheidea, Astacidea, Caridea, and Penaeoidea. The infraorder Axiidea was moderately supported. Support values decreased towards the root of the tree (Fig. 4). Still, several nodes located near the root of the phylogenetic tree were well supported (Supplementary Fig. S3). The above suggests that mitochondrial genomes alone will likely have enough phylogenetic information to reveal relationships at higher taxonomic levels within the Pancrustacea and Arthropoda.

## Conclusions

In conclusion, this study assembled for the first time the mitochondrial genome of the Caribbean spiny lobster *P*. *argus*, a keystone species in shallow water coral reefs^6,47^ and target of the most lucrative fishery in the greater Caribbean region^2^. The complete mitochondrial genome of the Caribbean spiny lobster *P*. *argus* will contribute to the better understanding of meta-population connectivity in this overexploited species. Sequencing of the whole genome of *P*. *argus* is underway.

## Electronic supplementary material

Supplementary Materials

## Data Availability

Data is available at Genebank (accession number MH068821).
