# Supplementary material for: The complete mitochondrial genome of the Caribbean spiny lobster Panulirus argus
Source: Sci Rep. 2018 Dec 6;8:17690. doi: 10.1038/s41598-018-36132-6 (PMC6283867; doi:10.1038/s41598-018-36132-6)
Supplement: Supplementary file 1 — Supplementary Materials [file 41598_2018_36132_MOESM1_ESM.pdf]

The complete mitochondrial genome of the Caribbean spiny lobster *Panulirus argus*: purifying selection and phylogenomic informativeness of protein coding genes.

J. Antonio Baeza[1, 2, 3,*]

[1] *Department of Biological Sciences, 132 Long Hall, Clemson University, Clemson, SC 29634, USA.*

[2] *Smithsonian Marine Station at Fort Pierce , 701 Seaway Drive, Fort Pierce, Florida 34949, USA.*

[3] *Departamento de Biología Marina, Facultad de Ciencias del Mar, Universidad Católica del Norte, Larrondo 1281, Coquimbo, Chile.*

[*]corresponding author: JA Baeza, jbaezam@clemson.edu, phone:1 772 475 0053

Supplementary Materials

Supplementary Table S1. Species names, mitochondrial genome sequences, and GenBank numbers used for the ML phylogenetic analysis conducted in MITOPhast.

Species	Accession ID
**Crustacea DECAPODA PLEOCYEMATA**	
**ACHELATA (infraorder)**	
Palinurellus wieneckii	KC847078
Panulirus homarus	JN542716
Panulirus japonicus	AB071201
Panulirus ornatus	GQ223286
Panulirus stimpsoni	GQ292768
Panulirus versicolor	KC107808
Sagmariasus verreauxi	AB859775
Scyllarides latus	KC107814
**ANOMURA (infraorder)**	
Cryptolithodes sitchensis	KC107820

Lithodes nintokuae	AB769476
Neopetrolisthes maculatus	KC107816
Pagurus longicarpus	AF150756
Paralithodes brevipes	AB735677
Shinkaia crosnieri	EU420129
**ASTACIDEA (infraorder)**	
Cambaroides similis	JN991196
Cherax quadricarinatus	KF649850
Enoplometopus occidentalis	KC107819
Euastacus yarraensis	HG942176
Homarus americanus	HQ402925
Procambarus fallax	KC107813
**AXIIDEA (infraorder)**	
Calocaris macandreae	KC107812
Callianassa ceramica	KU350630.1
Corallianassa coutierei	KC107817
Nihonotrypaea japonica	KC236422
Nihonotrypaea thermophila	JN897380
Neaxius acanthus	KC107821
Neaxius glyptocercus	JN897379
Trypaea australiensis	KM501040.2
**BRACHYURA (infraorder)**	
Chaceon granulatus	AB769383
Charybdis japonica	FJ460517
Gandalfus yunohana	EU647222
Geothelphusa dehaani	AB187570
Pseudocarcinus gigas	AY562127
Ranina ranina	AB752308
**CARIDEA (infraorder)**	
Alpheus distinguendus	GQ892049
Exopalaemon carinicauda	EF560650
Halocaridina rubra	DQ917432
Macrobrachium rosenbergii	AY659990
Nautilocaris saintlaurentae	KF226726
Neocaridina denticulata	JX156333
**GEEBIDAE (infraorder)**	
Austinogebia edulis	JN897376
Thalassina kelanang	JN897378

Upogebia major	JN897377
Upogebia pusilla	KC107815
**GLYPHEIDEA (infraorder)**	
Laurentaeglyphea neocaledonica	*KU500619*
Neoglyphea inopinata	*KT984196*
**POLYCHELIDAE (infraorder)**	
Polycheles typhlops	KC107818
**STENOPODIDAE (infraorder)**	
Stenopus hispidus	JN399096
**Crustacea DECAPODA DENDROBRANCHIATA**	
**PENAEOIDEA (superfamily)**	
Farfantepenaeus californiensis	EU497054
Fenneropenaeus chinensis	DQ518969
Litopenaeus vannamei	EF584003
Penaeus monodon	AF217843
Acetes chinensis	JN689221
**Crustacea DECAPODA HOPLOCARIDA**	
**STOMATOPODA (order)**	
Gonodactylus chiragra	DQ191682
Lysiosquillina maculata	DQ191683
Harpiosquilla harpax^{+}	AY699271
Squilla mantis^{+}	AY639936
**Crustacea PERACARIDA**	
**AMPHIPODA (order)**	
Caprella scaura	NC_014687
Metacrangonyx repens	NC_019653
Gondogeneia antarctica	NC_016192
Pseudoniphargus daviui	NC_019662
Gammarus duebeni	NC_017760
Onisimus nanseni	NC_013819
Bahadzia jaraguensis	NC_019661
Platorchestia parapacifica	MG010371
Eurythenes maldoror	MF766257
**ISOPODA (order)**	
Ligia oceanica	NC_008412
Eophreatoicus sp.14	NC_013976

**MYSIDA (order)**	
Neomysis orientalis	KC995119
Neomysis japonica	KR006340
**Crustacea THECOSTRACA**	
Capitulum mitela	AY514042
Tetraclita rufotincta	KY865100
Altiverruca navicula	MG252956
Tesseropora rosea	KY865099
Eochionelasmus ohtai	MF939636
Epopella plicata	KM008743
Pollicipes pollicipes	FJ798974
Chelonibia testudinaria	KJ754819
Tetraclitella divisa	KJ754822
Acasta sulcata	NC_029168
Armatobalanus allium	KJ754817
Octomeris sp.	KJ754820
Savignium sp.	KJ754821
Chthamalus antennatus	KP294312
Lepas anserifera	NC_026576
Balanus balanus	KM660676
Megabalanus ajax	KF501046
Amphibalanus amphitrite	KF588709
Notochthamalus scabrosus	KF425565
**BRANCHIOPODA**	
Triops longicaudatus	GU475465
Daphnia melanica	DQ340845
Phallocryptus tserensodnomi	KP273592
Streptocephalus sirindhornae	KP273593
Artemia franciscana	NC_001620
**CEPHALOCARIDA**	
Hutchinsoniella macracantha	AY456189
**REMIPEDIA**	
Speleonectes tulumensis	AY456190
**COPEPODA**	
Calanus sinicus	GU355641
Paracyclopina nana	EU877959
Sinergasilus polycorpus	KR263117

Pandarus rhincodonicus	MF872726
Onisimus nanseni	FJ555185
Lernaea cyprinacea	KM235194
Tigriopus japonicus	AY959338
Lepeophtheirus salmonis	AY625897
**BRANCHIURA**	
Argulus americanus	AY456187
**OSTRACODA**	
Cypridopsis vidua	KP063117
Vargula hilgendorfii	AB114300
**PENTASTOMIDA**	
Armillifer armillatus	AY456186
Armillifer agkistrodontis	KX686568
**COLLEMBOLA**	
Folsomotoma octooculata	KC862316
Cryptopygus antarcticus	NC_010533
Folsomia candida	KU198392
Orchesella cincta	KT985987
Friesea grisea	EU124719
Bilobella aurantiaca	EU084034
Sminthurus viridis	EU016192
Orchesella villosa	EU016195
Podura aquatica	AY639939
Onychiurus orientalis	AY639938
Gomphiocephalus hodgsoni	AY191995
Tetrodontophora bielanensis	AF272824
Onychiurus orientalis	NC_006074
**INSECTA**	
Davidius lunatus	EU591677
Rhopaea magnicornis	FJ859903
Petrobius brevistylis	AY956355
Pedetontus silvestrii	EU621793
Gomphocerippus rufus	GU294759
Atelura formicaria	EU084035
Thermobia domestica	AY639935
Tricholepidion gertschi	AY191994
Atelura formicaria	EU084035

**MYRIAPODA**	
Scutigerella causeyae	DQ666065
Symphylella sp.	EF576853
Sphaerotheriidae sp.	JQ713564
Pauropus longiramus	HQ457012
Glomeridesmus spelaeus	MG372113
Anaulaciulus koreanus	KX096886
Xystodesmus sp.	KU721886
Asiomorpha coarctata	KU721885
Antrokoreana gracilipes	DQ344025
Brachycybe lecontii	JX437064
Appalachioria falcifera	JX437063
Abacion magnum	JX437062
Strigamia maritima	KP173664
Scolopocryptops sp. 1	KC200076
Cermatobius longicornis	KC155628
Narceus annularus	AY055727
Thyropygus sp.	AY055728
Narceus annularus	AY055727
Bothropolys sp.	AY691655
Scutigera coleoptrata	NC_005870
Lithobius forficatus	AF309492
Epiperipatus biolleyi	DQ666064
**CHELICERATA**	
Limulus polyphemus	JX983598
**EUPHAUSIACEA**	
Euphausia pacifica	EU587005

Supplementary Table S2. Species by gene presence matrix used in the 'Total evidence' phylogenetic tree obtained from ML analysis of complete PCGs for pancrustaceans and other selected taxa from the phylum Arthropoda. Gene order in the matrix is 1=TP6, 2=ATP, 3=COB, 4=COX1, 5=COX2, 6=COX3, 7=NAD1, 8=NAD2, 9=NAD3, 10=NAD4, 11=NAD4L, 12=NAD5, and 13=NAD6. Gene presence in the data matrix is indicated with 'y' while gene absence is indicated with '**n**'

**Species x Gene Presence**	**1**	**2**	**3**	**4**	**5**	**6**	**7**	**8**	**9**	**10**	**11**	**12**	**13**
**ACHELATA (infraorder)**													
Palinurellus wieneckii	y	y	y	y	y	y	y	y	y	y	y	y	y
Panulirus homarus	y	y	y	y	y	y	y	y	y	y	y	y	y
Panulirus japonicus	y	y	y	y	y	y	y	y	y	y	y	y	y
Panulirus ornatus	y	y	y	y	y	y	y	y	y	y	y	y	y
Panulirus stimpsoni	y	y	y	y	y	y	y	y	y	y	y	y	y
Panulirus versicolor	y	y	y	y	y	y	y	y	y	y	y	y	y
Sagmariasus verreauxi	y	y	y	y	y	y	y	y	y	y	y	y	y
Scyllarides latus	y	y	y	y	y	y	y	y	y	y	y	y	y
**ANOMURA (infraorder)**													
Cryptolithodes sitchensis	y	y	y	y	y	y	**n**	y	y	y	y	y	y
Lithodes nintokuae	y	y	y	y	y	y	y	y	y	y	y	y	y
Neopetrolisthes maculatus	y	y	y	y	y	y	y	y	y	y	y	y	y
Pagurus longicarpus	y	y	y	y	y	y	y	y	y	y	y	y	y
Paralithodes brevipes	y	y	y	y	y	y	y	y	y	y	y	y	y
Shinkaia crosnieri	y	y	y	y	y	y	y	y	y	y	y	y	y
**ASTACIDEA (infraorder)**													
Cambaroides similis	y	y	y	y	y	y	y	y	y	y	y	y	y
Cherax quadricarinatus	y	y	y	y	y	y	y	y	y	y	y	y	y
Enoplometopus occidentalis	y	y	y	y	y	y	y	**n**	y	y	y	y	y
Euastacus yarraensis	y	y	y	y	y	y	y	y	y	y	y	y	y
Homarus americanus	y	y	y	y	y	y	y	y	y	y	y	y	y
Procambarus fallax	y	y	y	y	y	y	y	y	y	y	y	y	y

**AXIIDEA (infraorder)**													
Calocaris macandreae	y	y	y	y	y	y	y	y	y	y	y	y	y
Callianassa ceramica	y	y	y	y	y	y	y	y	y	y	y	y	y
Corallianassa coutierei	y	y	y	y	y	y	y	y	y	y	y	y	y
Nihonotrypaea japonica	y	y	y	y	y	y	y	y	y	y	y	y	y
Nihonotrypaea thermophila	y	y	y	y	y	y	y	y	y	y	y	y	y
Neaxius acanthus	y	y	y	y	y	y	y	**n**	y	y	y	y	y
Neaxius glyptocercus	y	y	y	y	y	y	y	y	y	y	y	y	y
Trypaea australiensis	y	y	y	y	y	y	y	y	y	y	y	y	y
**BRACHYURA (infraorder)**													
Chaceon granulatus	y	y	y	y	y	y	y	y	y	y	y	y	y
Charybdis japonica	y	y	y	y	y	y	y	y	y	y	y	y	y
Gandalfus yunohana	y	y	y	y	y	y	y	y	y	y	y	y	y
Geothelphusa dehaani	y	y	y	y	y	y	y	y	y	y	y	y	y
Pseudocarcinus gigas	y	y	y	y	y	y	y	y	y	y	y	y	y
Ranina ranina	y	y	y	y	y	y	y	y	y	y	y	y	y
**CARIDEA (infraorder)**	y	y	y	y	y	y	y	y	y	y	y	y	y
Alpheus distinguendus	y	y	y	y	y	y	y	y	y	y	y	y	y
Exopalaemon carinicauda	y	y	y	y	y	y	y	y	y	y	y	y	y
Halocaridina rubra	y	y	y	y	y	y	y	y	y	y	y	y	y
Macrobrachium rosenbergii	y	y	y	y	y	y	y	y	y	y	y	y	y
Nautilocaris saintlaurentae	y	y	y	y	y	y	y	y	y	y	y	y	y
Neocaridina denticulata	y	y	y	y	y	y	y	y	y	y	y	y	y
**GEEBIDAE (infraorder)**													
Austinogebia edulis	y	y	y	y	y	y	y	y	y	y	y	y	y
Thalassina kelanang	y	y	y	y	y	y	y	y	y	y	y	y	y
Upogebia major	y	y	y	y	y	y	y	y	y	y	y	y	y
Upogebia pusilla	y	y	y	y	y	y	y	y	y	y	y	y	y
**GLYPHEIDEA (infraorder)**													
Laurentaeglyphea neocaledonica	y	y	y	y	y	y	y	y	y	y	y	y	y
Neoglyphea inopinata	y	y	y	y	y	y	y	y	y	y	y	y	y
**POLYCHELIDAE (infraorder)**													
Polycheles typhlops	y	y	y	y	y	y	y	y	y	y	y	y	y
**STENOPODIDAE (infraorder)**													

Stenopus hispidus	y	y	y	y	y	y	y	y	y	y	y	y	y
**PENAEOIDEA (superfamily)**													
Farfantepenaeus californiensis	y	y	y	y	y	y	y	y	y	y	y	y	y
Fenneropenaeus chinensis	y	y	y	y	y	y	y	y	y	y	y	y	y
Litopenaeus vannamei	y	y	y	y	y	y	y	y	y	y	y	y	y
Penaeus monodon	y	y	y	y	y	y	y	y	y	y	y	y	y
Acetes chinensis	y	y	y	y	y	y	y	y	y	y	y	y	y
**STOMATOPODA (order)**													
Gonodactylus chiragra	y	y	y	y	y	y	y	y	y	y	y	y	y
Lysiosquillina maculata	y	y	y	y	y	y	y	y	y	y	y	y	y
Harpiosquilla harpax[+]	y	y	y	y	y	y	y	y	y	y	y	y	y
Squilla mantis[+]	y	y	y	y	y	y	y	y	y	y	y	y	y
**AMPHIPODA (order)**													
Caprella scaura	y	y	y	y	y	y	y	y	y	y	y	y	y
Metacrangonyx repens	y	y	y	y	y	y	y	y	y	y	y	y	y
Gondogeneia antarctica	y	y	y	y	y	y	y	y	y	y	y	y	y
Pseudoniphargus daviui	y	y	y	y	y	y	y	y	y	y	y	y	y
Gammarus duebeni	y	y	y	y	y	y	y	y	y	y	y	y	y
Onisimus nanseni	y	y	y	y	y	y	y	y	y	y	y	y	y
Bahadzia jaraguensis	y	y	y	y	y	y	y	y	y	y	y	y	y
Platorchestia parapacifica	y	y	y	y	y	y	y	y	y	y	y	y	y
Eurythenes maldoror	y	y	y	y	y	y	y	y	y	y	y	y	y
**ISOPODA (order)**													
Ligia oceanica	y	y	y	y	y	y	y	y	y	y	y	y	y
Eophreatoicus sp.14	y	y	y	y	y	y	y	y	y	y	y	y	y
**MYSIDA (order)**													
Neomysis orientalis	y	y	y	y	y	y	y	y	y	y	y	y	y
Neomysis japonica	y	y	y	y	y	y	y	y	y	y	y	y	y
**Crustacea THECOSTRACA**													
Capitulum mitela	y	y	y	y	y	y	y	y	y	y	y	y	y
Tetraclita rufotincta	y	y	y	y	y	y	y	y	y	y	y	y	y
Altiverruca navicula	y	y	y	y	y	y	y	y	y	y	y	y	y
Tesseropora rosea	y	y	y	y	y	y	y	y	y	y	y	y	y
Eochionelasmus ohtai	y	y	y	y	y	y	y	y	y	y	y	y	y
Epopella plicata	y	y	y	y	y	y	y	y	y	y	y	y	y
Pollicipes pollicipes	y	y	**n**	y	y	y	**n**	y	y	**n**	**n**	**n**	**n**

Chelonibia testudinaria	y	y	y	y	y	y	y	y	y	y	y	y	y
Tetraclitella divisa	y	y	y	y	y	y	y	y	y	y	y	y	y
Acasta sulcata	y	y	y	y	y	y	y	y	y	y	y	y	y
Armatobalanus allium	y	y	y	y	y	y	y	y	y	y	y	y	y
Octomeris sp.	y	y	y	y	y	y	y	y	y	y	y	y	y
Savignium sp.	y	y	y	y	y	y	y	y	y	y	y	y	y
Chthamalus antennatus	y	y	y	y	y	y	y	y	y	y	y	y	y
Lepas anserifera	y	y	y	y	y	y	y	y	y	y	y	y	y
Balanus balanus	y	y	y	y	y	y	y	y	y	y	y	y	y
Megabalanus ajax	y	y	y	y	y	y	y	y	y	y	y	y	y
Amphibalanus amphitrite	y	y	y	y	y	y	y	y	y	y	y	y	y
Notochthamalus scabrosus	y	y	y	y	y	y	y	y	y	y	y	y	y
**BRANCHIOPODA**													
Triops longicaudatus	y	y	y	y	y	y	y	y	y	y	y	y	y
Daphnia melanica	y	y	y	y	y	y	y	y	y	y	y	y	y
Phallocryptus tserensodnomi	y	y	y	y	y	y	y	y	y	y	y	y	y
Streptocephalus sirindhornae	y	y	y	y	y	y	y	y	y	y	y	y	y
Artemia franciscana	y	y	y	y	y	y	y	y	y	y	y	y	y
**CEPHALOCARIDA**													
Hutchinsoniella macracantha	y	y	y	y	y	y	y	y	y	y	y	y	y
**REMIPEDIA**													
Speleonectes tulumensis	y	y	y	y	y	y	y	y	y	y	y	y	y
**COPEPODA**													
Calanus sinicus	y	y	y	y	y	y	y	y	y	y	y	y	y
Paracyclopina nana	y	**n**	y	y	y	y	y	y	y	y	y	y	y
Sinergasilus polycorpus	y	y	y	y	y	y	y	y	y	y	y	y	y
Pandarus rhincodonicus	y	y	y	y	y	y	y	y	y	y	y	y	y
Onisimus nanseni	y	y	y	y	y	y	y	y	y	y	y	y	y
Lernaea cyprinacea	y	y	y	y	y	y	y	y	y	y	y	y	y
Tigriopus japonicus	y	y	y	y	y	y	y	y	y	y	y	y	y
Lepeophtheirus salmonis	y	**n**	y	y	y	y	y	y	y	y	y	y	y
**BRANCHIURA**													
Argulus americanus	y	y	y	y	y	y	y	y	y	y	y	y	y
**OSTRACODA**													
Cypridopsis vidua	y	y	y	y	y	y	y	y	y	y	y	y	y
Vargula hilgendorfii	y	y	y	y	y	y	y	y	y	y	y	y	y

**PENTASTOMIDA**													
Armillifer armillatus	y	y	y	y	y	y	y	y	y	y	y	y	y
Armillifer agkistrodontis	y	y	y	y	y	y	y	y	y	y	y	y	y
**COLLEMBOLA**													
Folsomotoma octooculata	y	y	y	y	y	y	y	y	y	y	y	y	y
Cryptopygus antarcticus	y	y	y	y	y	y	y	y	y	y	y	y	y
Folsomia candida	y	y	y	y	y	y	y	y	y	y	y	y	y
Orchesella cincta	y	y	y	y	y	y	y	y	y	y	y	y	y
Friesea grisea	y	y	y	y	y	y	y	y	y	y	y	y	y
Bilobella aurantiaca	y	y	y	y	y	y	y	y	y	y	y	y	y
Sminthurus viridis	y	y	y	y	y	y	y	y	y	y	y	y	y
Orchesella villosa	y	y	y	y	y	y	y	y	y	y	y	y	y
Podura aquatica	y	y	y	y	y	y	y	y	y	y	y	y	y
Onychiurus orientalis	y	y	y	y	y	y	y	y	y	y	y	y	y
Gomphiocephalus hodgsoni	y	y	y	y	y	y	y	y	y	y	y	y	y
Tetrodontophora bielanensis	y	y	y	y	y	y	y	y	y	y	y	y	y
Onychiurus orientalis	y	y	y	y	y	y	y	y	y	y	y	y	y
**INSECTA**													
Davidius lunatus	y	y	y	y	y	y	y	y	y	y	y	y	y
Rhopaea magnicornis	y	y	y	y	y	y	y	y	y	y	y	y	y
Petrobius brevistylis	y	y	y	y	y	y	y	y	y	y	y	y	y
Pedetontus silvestrii	y	y	y	y	y	y	y	y	y	y	y	y	y
Gomphocerippus rufus	y	y	y	y	y	y	y	y	y	y	y	y	y
Atelura formicaria	y	y	y	y	y	y	y	y	y	y	y	y	y
Thermobia domestica	y	y	y	y	y	y	y	y	y	y	y	y	y
Tricholepidion gertschi	y	y	y	y	y	y	y	y	y	y	y	y	y
Atelura formicaria	y	y	y	y	y	y	y	y	y	y	y	y	y
**MYRIAPODA**													
Scutigerella causeyae	y	y	y	y	y	y	y	y	y	y	y	y	y
Symphylella sp.	y	y	y	y	y	y	y	y	y	y	y	y	y
Sphaerotheriidae sp.	y	y	y	y	y	y	y	y	y	y	y	y	y
Pauropus longiramus	y	y	y	y	y	y	y	y	y	y	y	y	y
Glomeridesmus spelaeus	y	y	y	y	y	y	y	y	y	y	y	y	y
Anaulaciulus koreanus	y	y	y	y	y	y	y	y	y	y	y	y	y
Xystodesmus sp.	y	y	y	y	y	y	y	y	y	y	y	y	y
Asiomorpha coarctata	y	y	y	y	y	y	y	y	y	y	y	y	y
Antrokoreana gracilipes	y	y	y	y	y	y	y	y	y	y	y	y	y
Brachycybe lecontii	y	y	y	y	y	y	y	y	y	y	y	y	y
Appalachioria falcifera	y	y	y	y	y	y	y	y	y	y	y	y	y
Abacion magnum	y	y	y	y	y	y	y	y	y	y	y	y	y

Strigamia maritima	y	y	y	y	y	y	y	y	y	y	y	y	y
Scolopocryptops sp. 1	y	y	y	y	y	y	y	y	y	y	y	y	y
Cermatobius longicornis	y	y	y	y	y	y	y	y	y	y	y	y	y
Narceus annularus	y	y	y	y	y	y	y	y	y	y	y	y	y
Thyropygus sp.	y	y	y	y	y	y	y	y	y	y	y	y	y
Narceus annularus	y	y	y	y	y	y	y	y	y	y	y	y	y
Bothropolys sp.	y	y	y	y	y	y	y	y	y	y	y	y	y
Scutigera coleoptrata	y	y	y	y	y	y	y	y	y	y	y	y	y
Lithobius forficatus	y	y	y	y	y	y	y	y	y	y	y	y	y
Epiperipatus biolleyi	y	y	y	y	y	y	y	y	y	y	y	y	y
**CHELICERATA**													
Limulus polyphemus	y	y	y	y	y	y	y	y	y	y	y	y	y
**EUPHAUSIACEA**													
Euphausia pacifica	y	y	y	y	y	y	y	y	y	y	y	y	y

Supplementary Figure S1. Selective pressure analysis in PCGs of *Panulirus argus*. K_A, K_S and K_A / K_S values were calculated using the γ-MYN model and adopting a sliding window of length = 52 and step length = 12. See methods and results for further details.

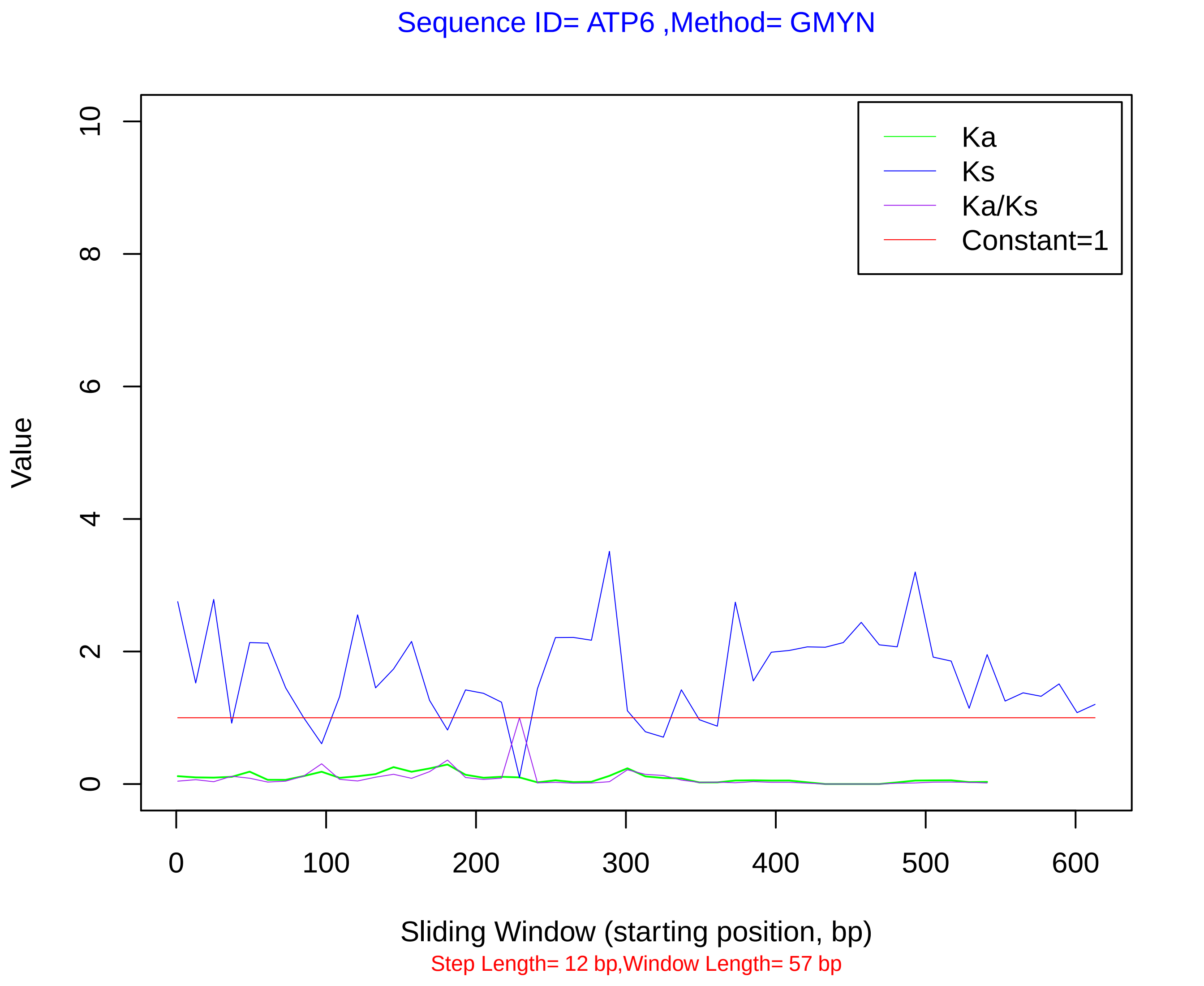

Sequence ID= ATP6 ,Method= GMYN
Ka
Ks
Ka/Ks
Constant=1
Value
0
2
4
6
8
10
0
100
200
300
400
500
600
Sliding Window (starting position, bp)
Step Length= 12 bp,Window Length= 57 bp

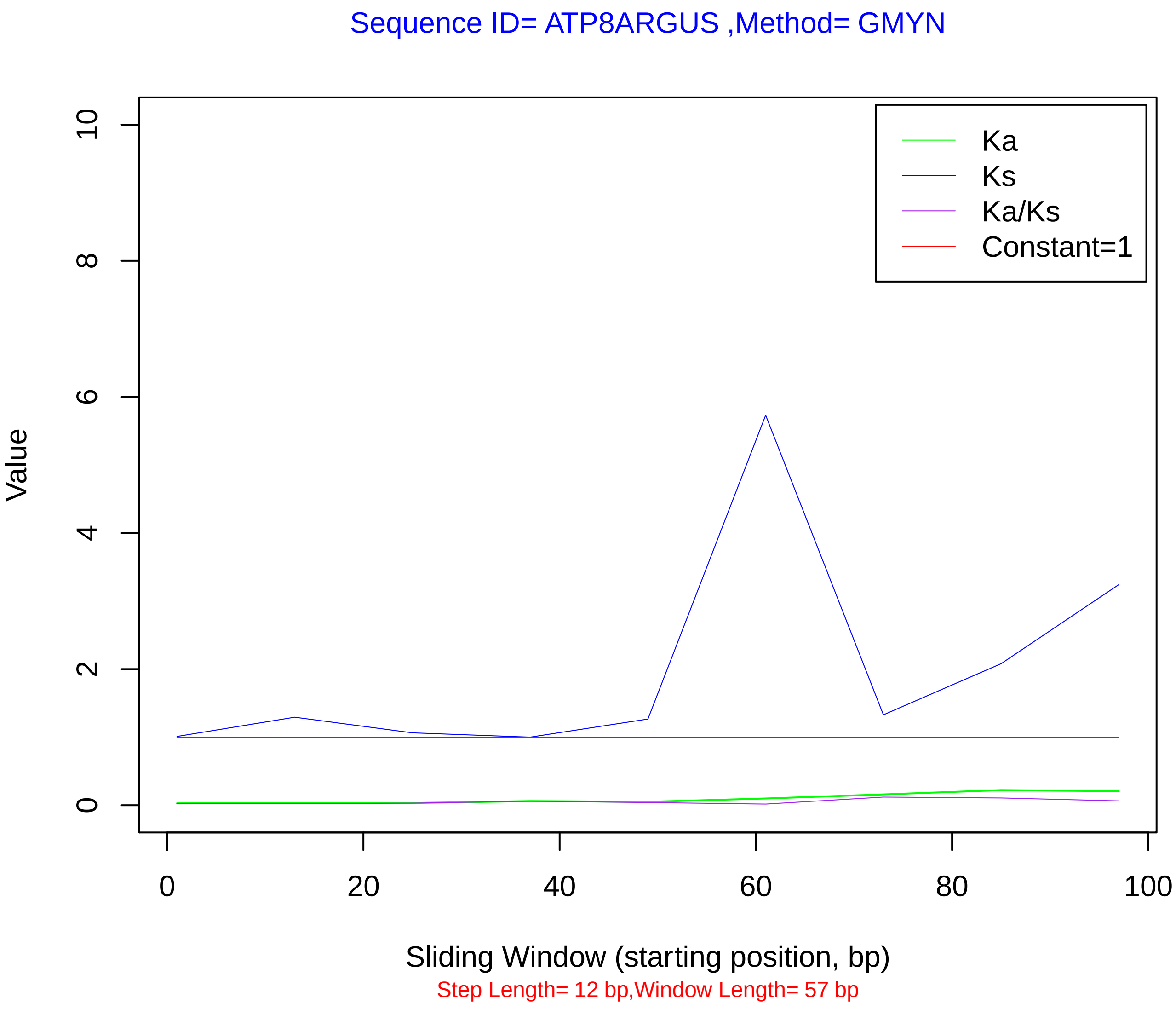

Sequence ID= ATP8ARGUS ,Method= GMYN
Ka
Ks
Ka/Ks
Constant=1
Value
0
2
4
6
8
10
0
20
40
60
80
100
Sliding Window (starting position, bp)
Step Length= 12 bp,Window Length= 57 bp

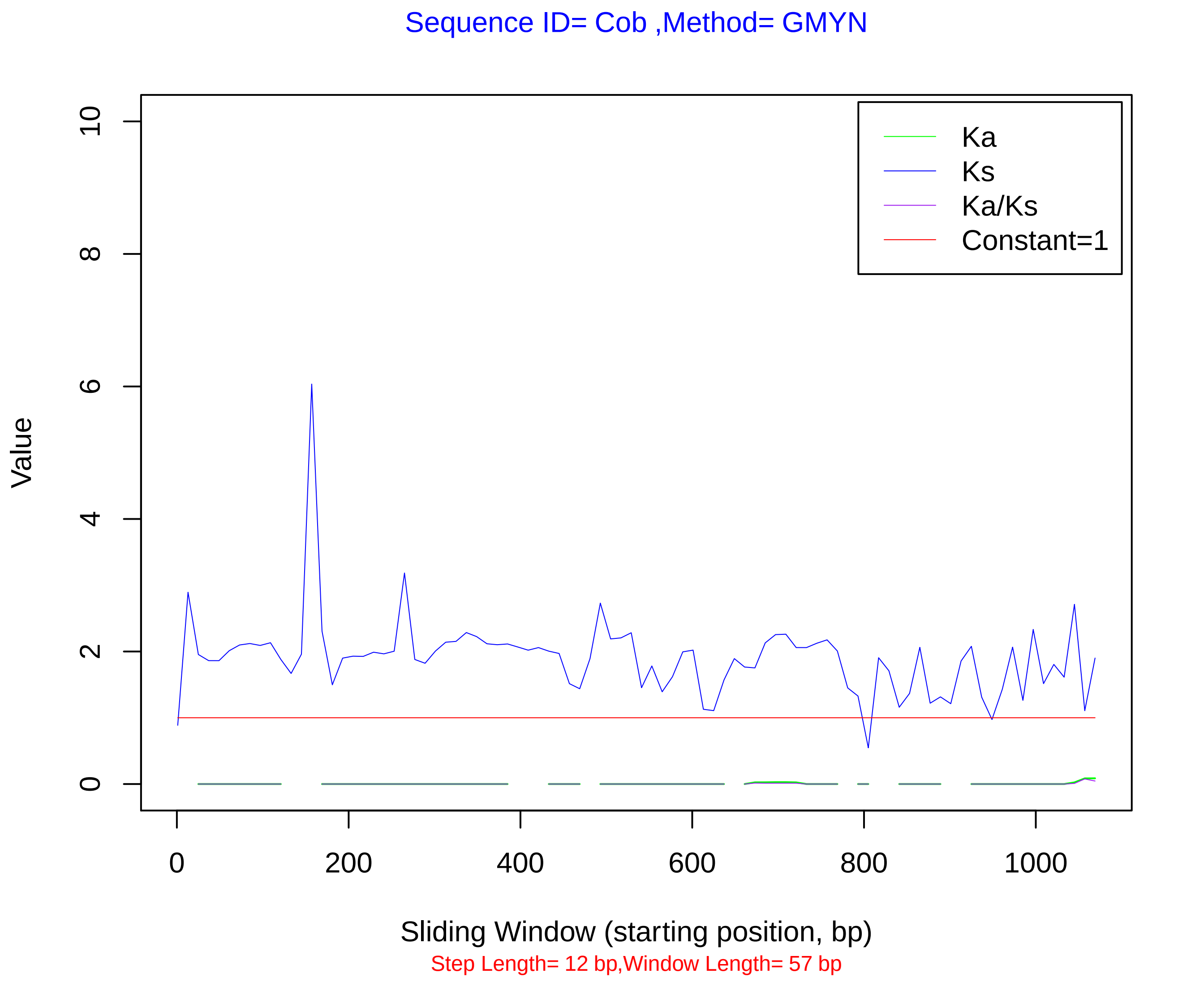

Sequence ID= Cob ,Method= GMYN
Value
0
2
4
6
8
10
0
200
400
600
800
1000
Ka
Ks
Ka/Ks
Constant=1
Sliding Window (starting position, bp)
Step Length= 12 bp,Window Length= 57 bp

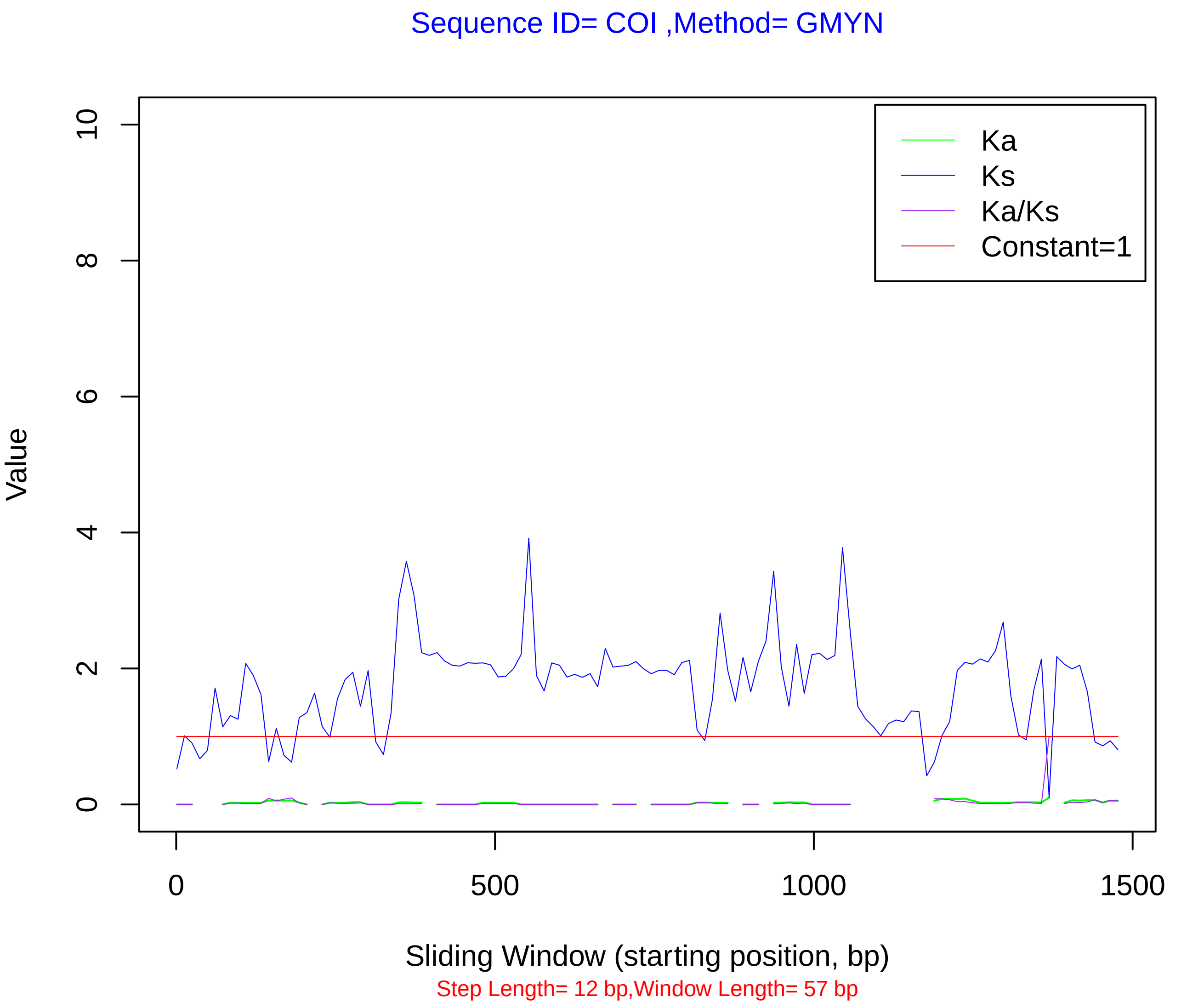

Sequence ID= COI ,Method= GMYN
Ka
Ks
Ka/Ks
Constant=1
Value
0
2
4
6
8
10
0
500
1000
1500
Sliding Window (starting position, bp)
Step Length= 12 bp,Window Length= 57 bp

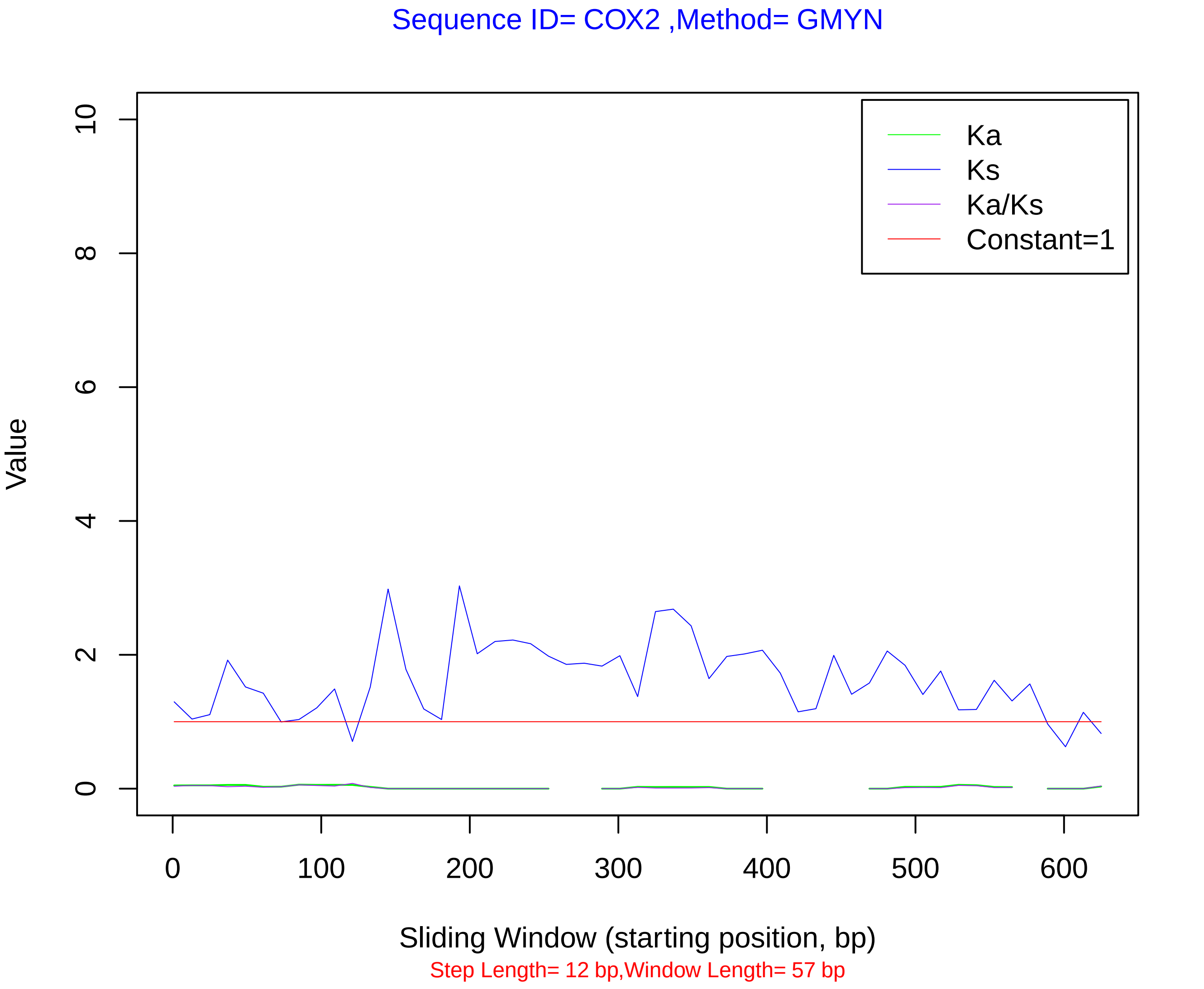
Sequence ID= COX2 ,Method= GMYN
Value
0
2
4
6
8
10
0
100
200
300
400
500
600
Ka
Ks
Ka/Ks
Constant=1
Sliding Window (starting position, bp)
Step Length= 12 bp,Window Length= 57 bp

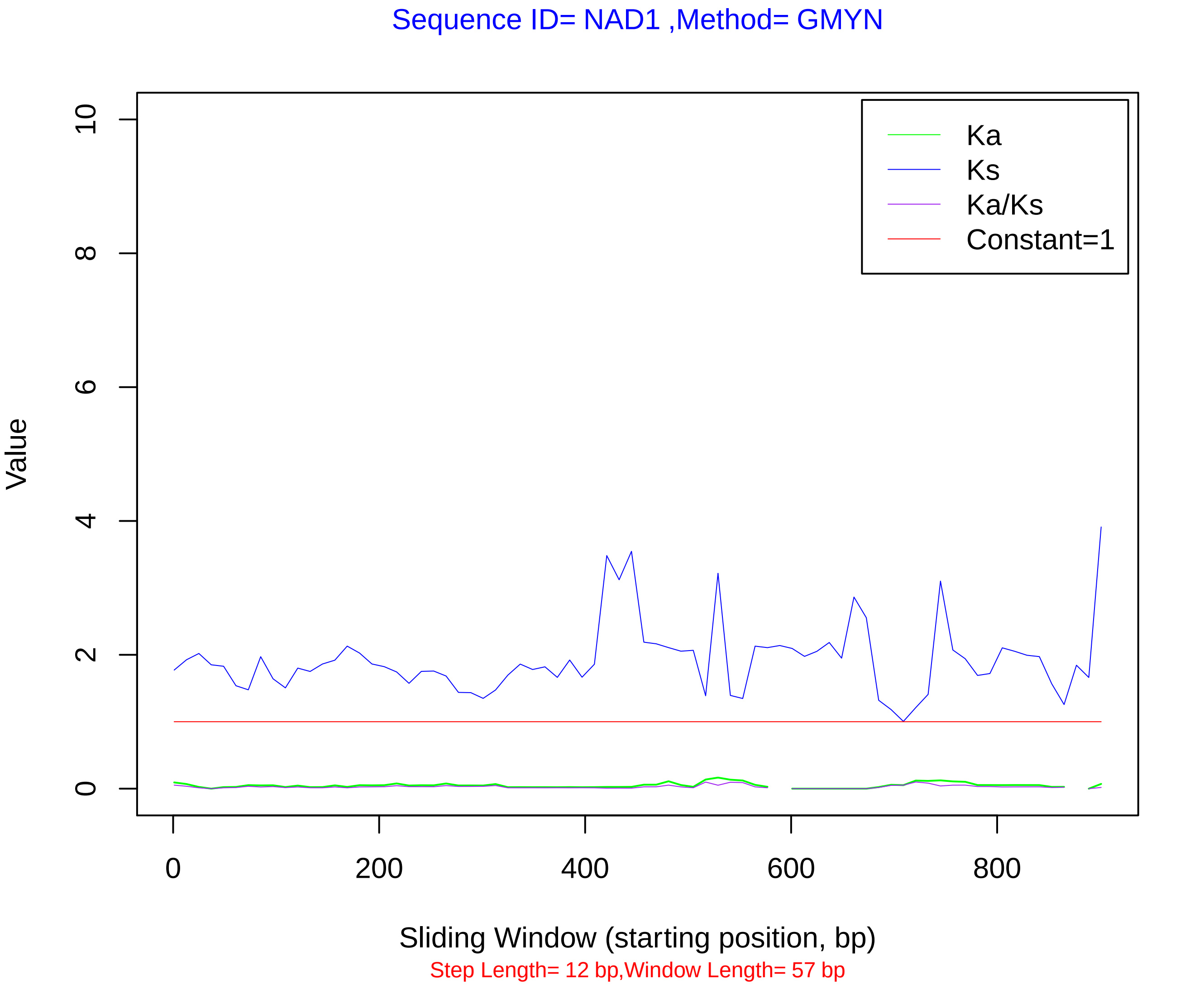

Sequence ID= NAD1 ,Method= GMYN
Ka
Ks
Ka/Ks
Constant=1
Value
0
2
4
6
8
10
0
200
400
600
800
Sliding Window (starting position, bp)
Step Length= 12 bp,Window Length= 57 bp

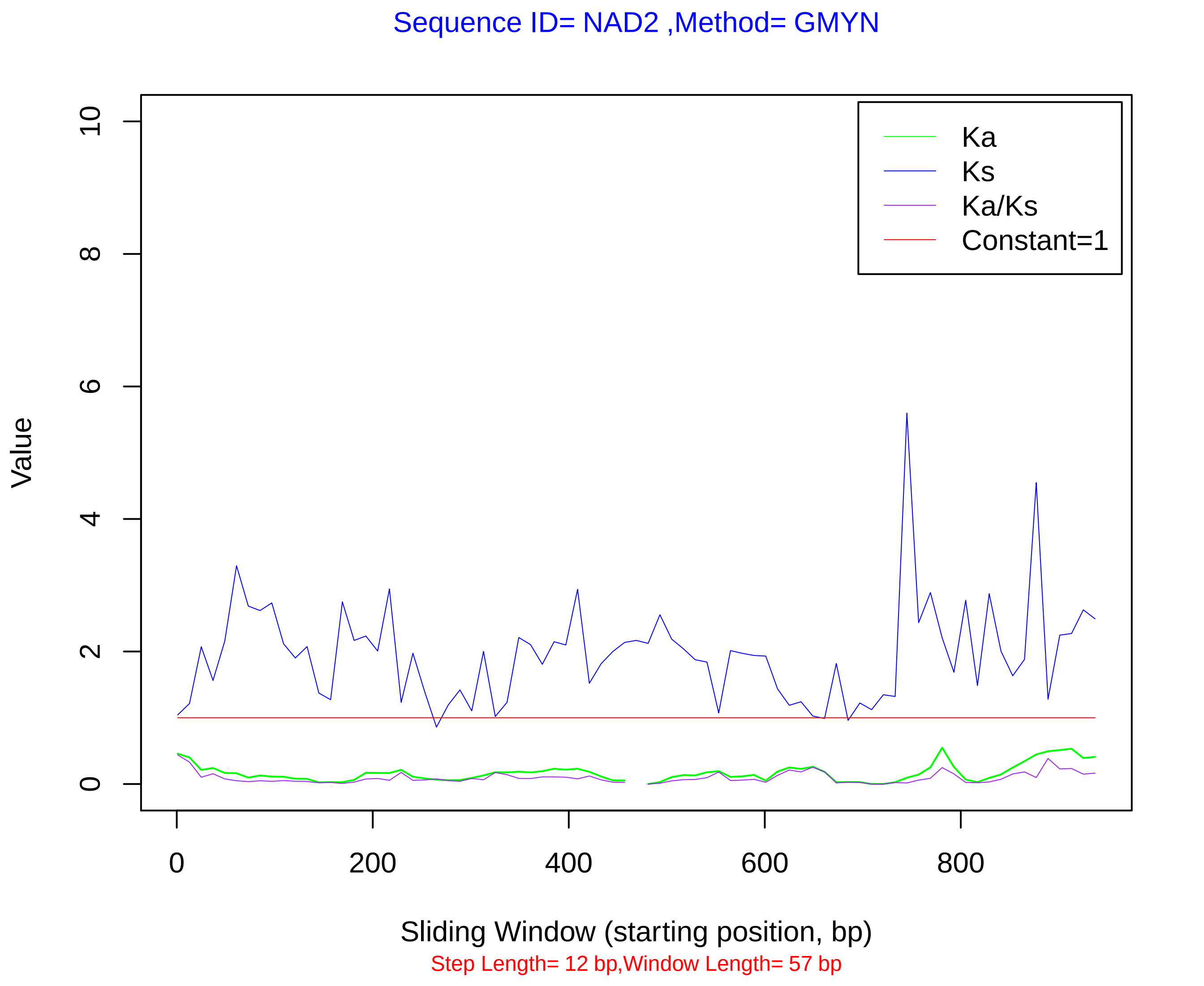
Sequence ID= NAD2 ,Method= GMYN
Value
0
2
4
6
8
10
0
200
400
600
800
Ka
Ks
Ka/Ks
Constant=1
Sliding Window (starting position, bp)
Step Length= 12 bp,Window Length= 57 bp

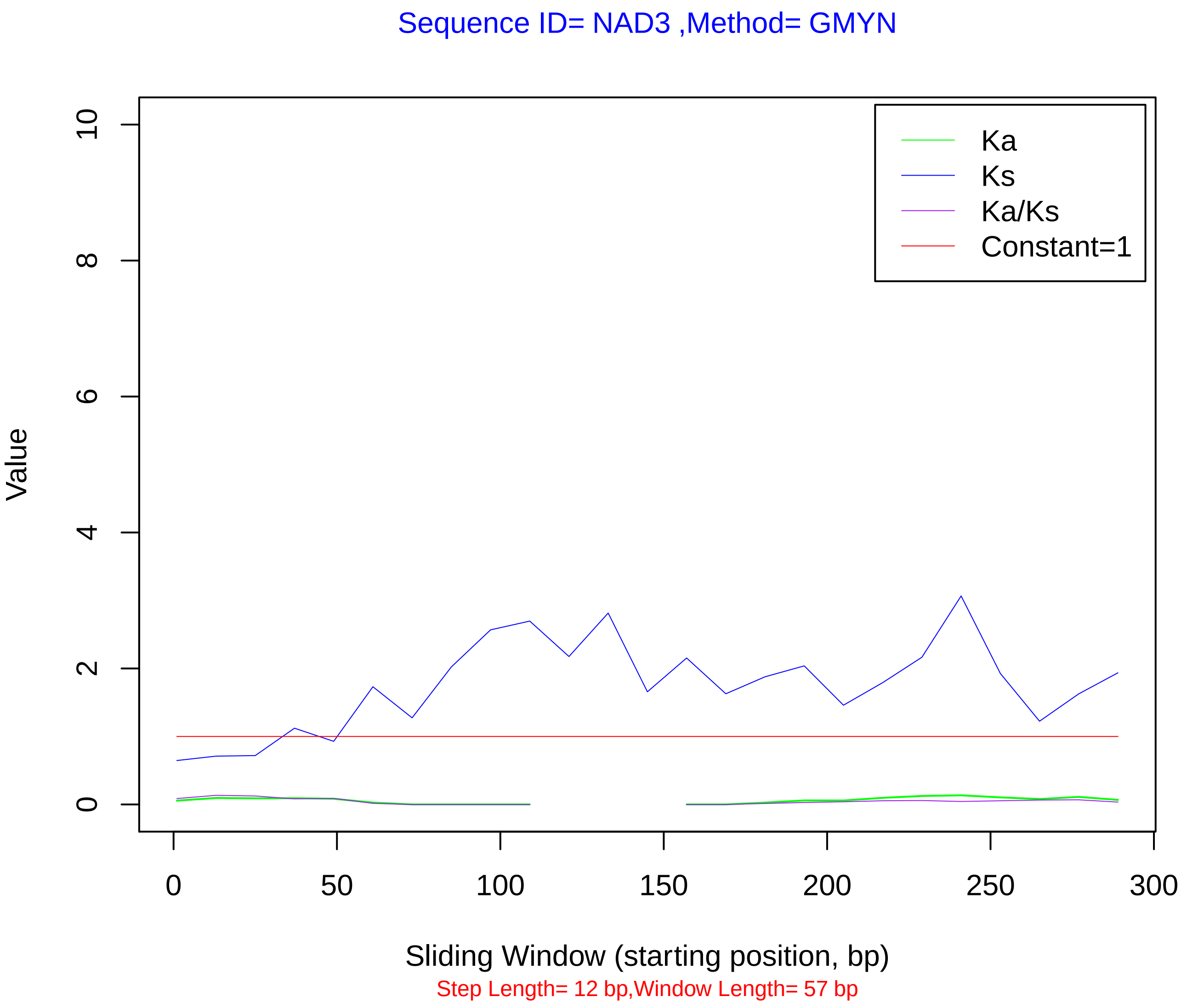
Sequence ID= NAD3 ,Method= GMYN
Ka
Ks
Ka/Ks
Constant=1
Value
0
2
4
6
8
10
0
50
100
150
200
250
300
Sliding Window (starting position, bp)
Step Length= 12 bp,Window Length= 57 bp

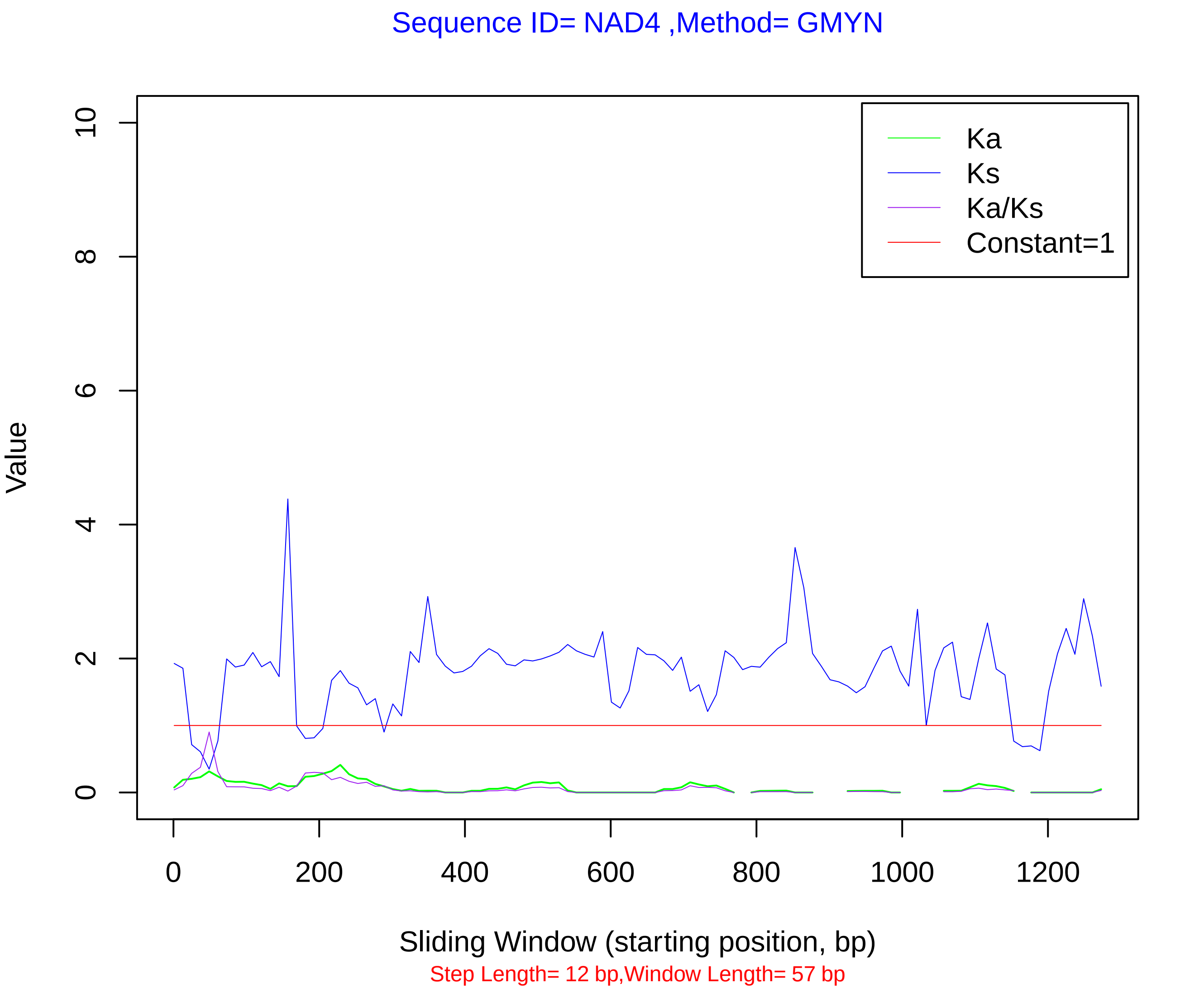

Sequence ID= NAD4 ,Method= GMYN
Ka
Ks
Ka/Ks
Constant=1
Value
0
2
4
6
8
10
0
200
400
600
800
1000
1200
Sliding Window (starting position, bp)
Step Length= 12 bp,Window Length= 57 bp

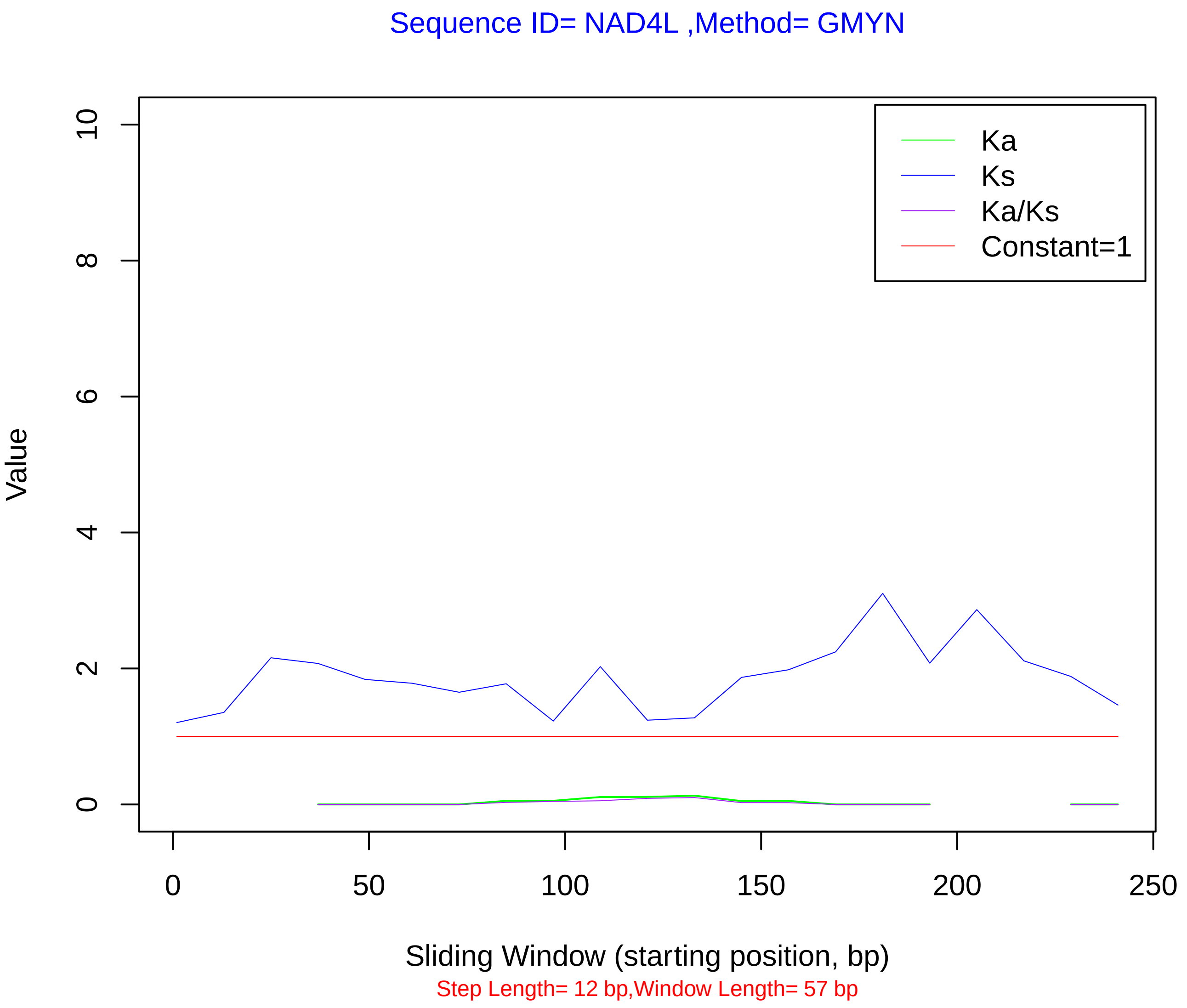
Sequence ID= NAD4L ,Method= GMYN
Ka
Ks
Ka/Ks
Constant=1
Value
0
2
4
6
8
10
0
50
100
150
200
250
Sliding Window (starting position, bp)
Step Length= 12 bp,Window Length= 57 bp

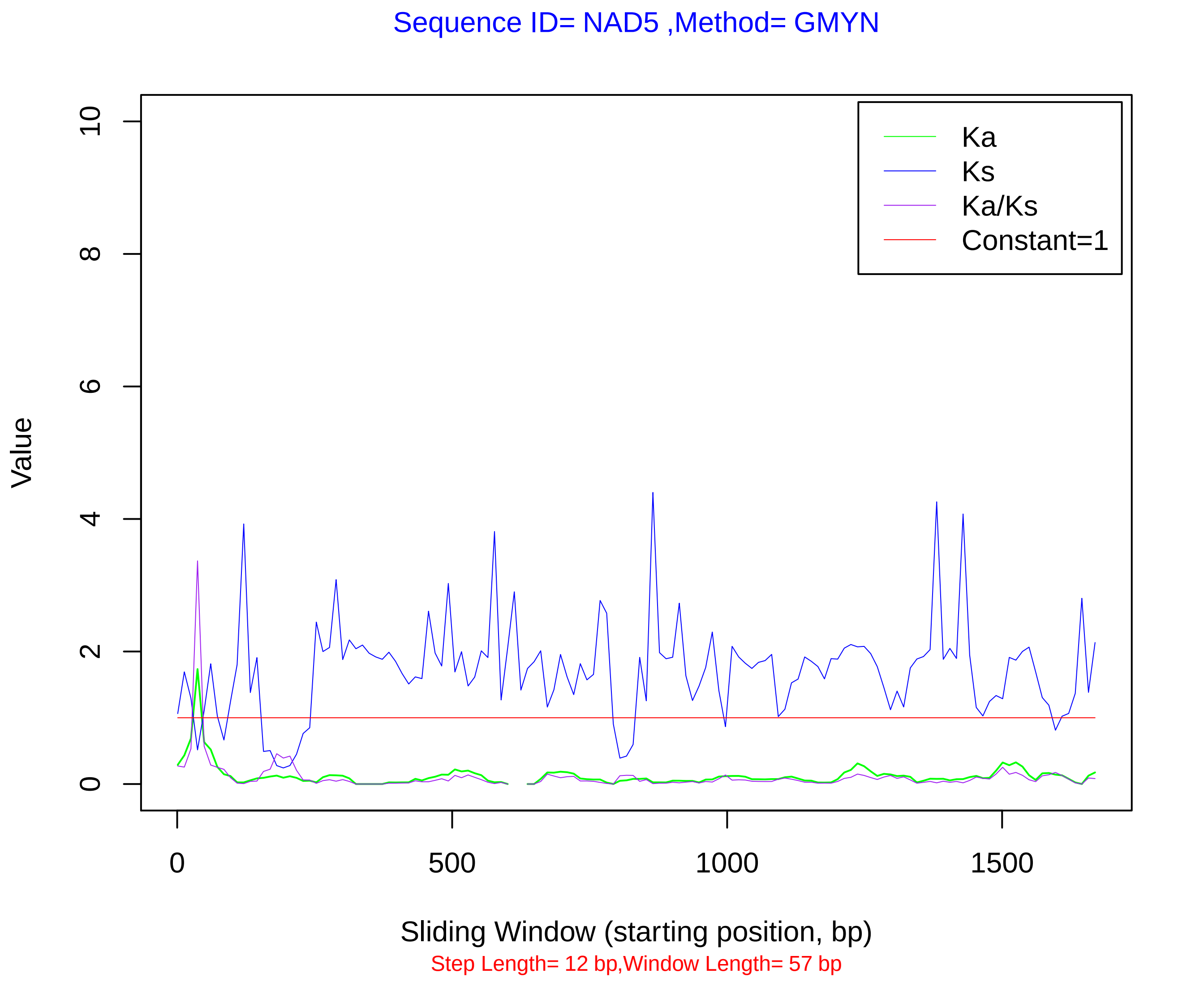

Sequence ID= NAD5 ,Method= GMYN
Value
0
2
4
6
8
10
0
500
1000
1500
Ka
Ks
Ka/Ks
Constant=1
Sliding Window (starting position, bp)
Step Length= 12 bp,Window Length= 57 bp

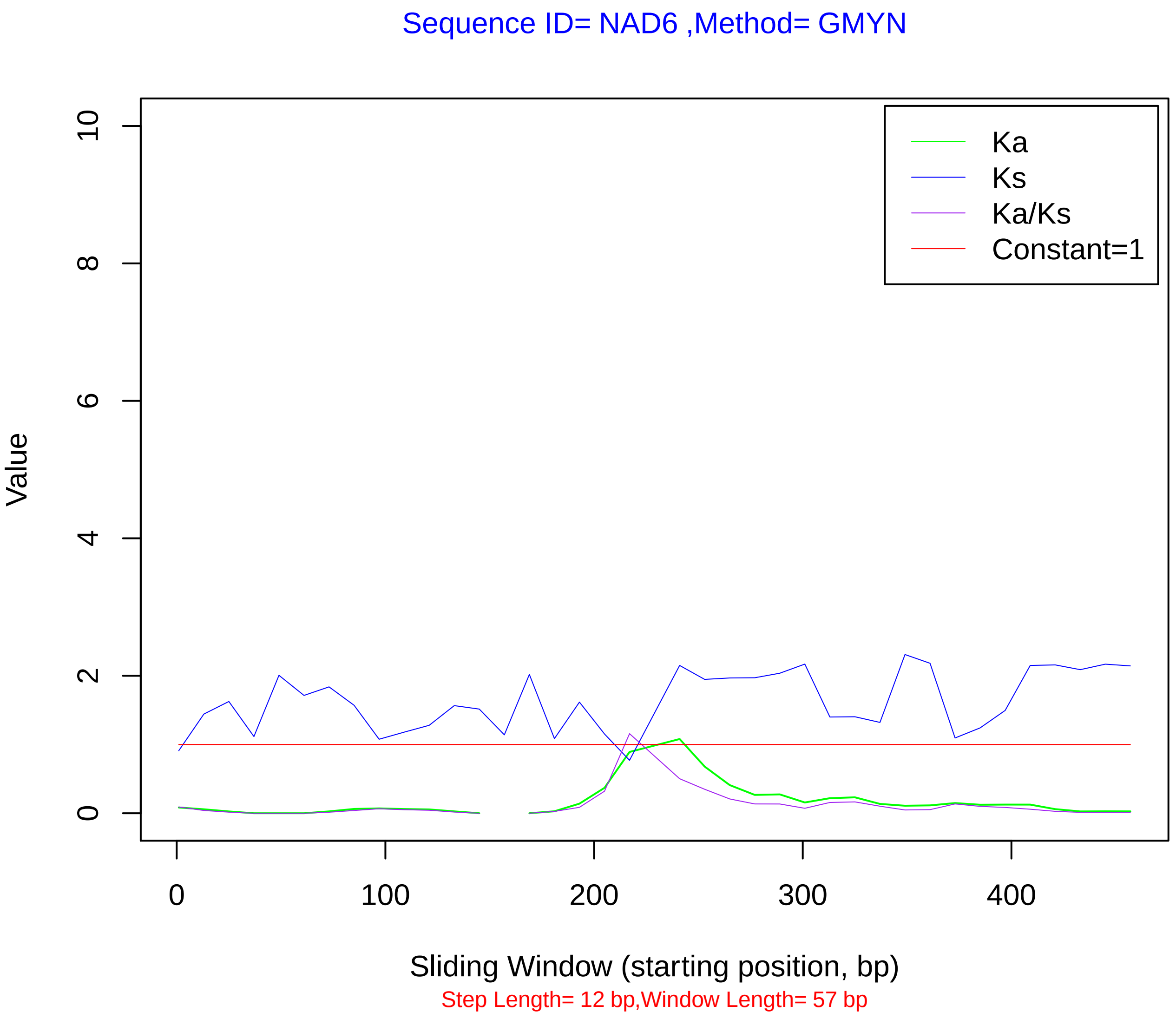
Sequence ID= NAD6 ,Method= GMYN
Ka
Ks
Ka/Ks
Constant=1
Value
0
2
4
6
8
10
0
100
200
300
400
Sliding Window (starting position, bp)
Step Length= 12 bp,Window Length= 57 bp

Supplementary Figure S2.

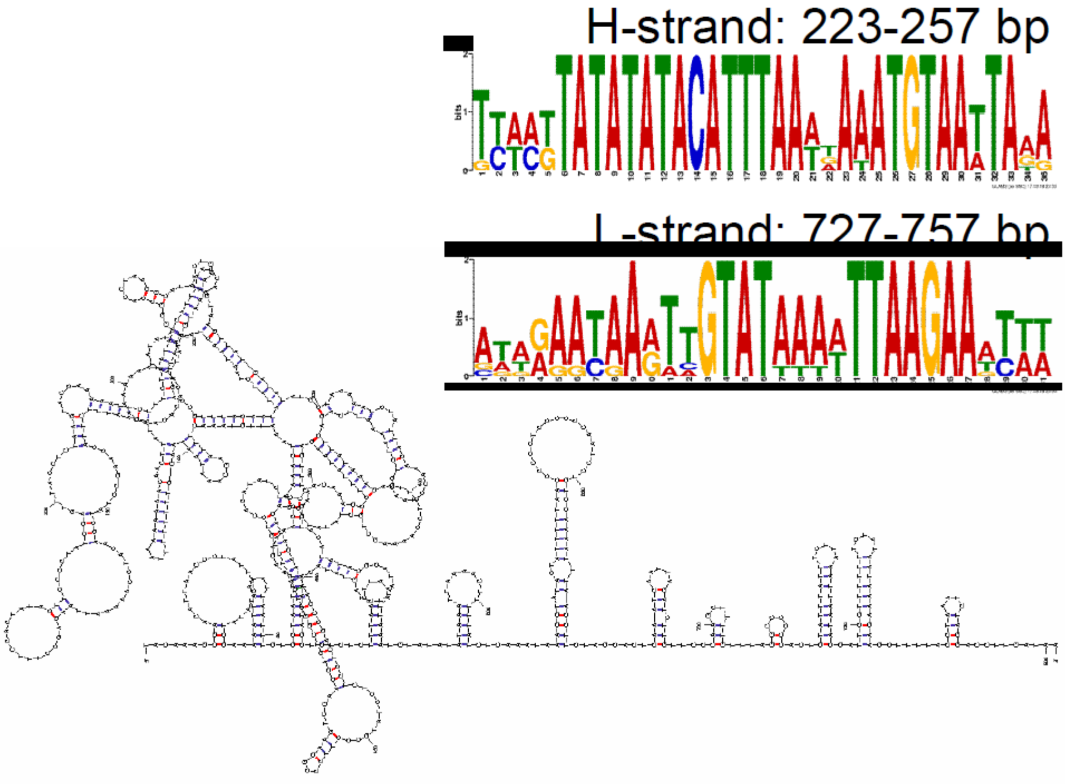

Supplementary Figure S2. GLAM2 recovered AT-rich motifs and secondary structure prediction analysis of the putative D-loop/CR in *Panulirus argus*.

Supplementary Figure S3. 'Total evidence' phylogenetic tree obtained from ML analysis of complete PCGs for pancrustaceans and other selected taxa from the phylum Arthropoda. Numbers above and/or below the branches represent the bootstrap values obtained from ML analysis.

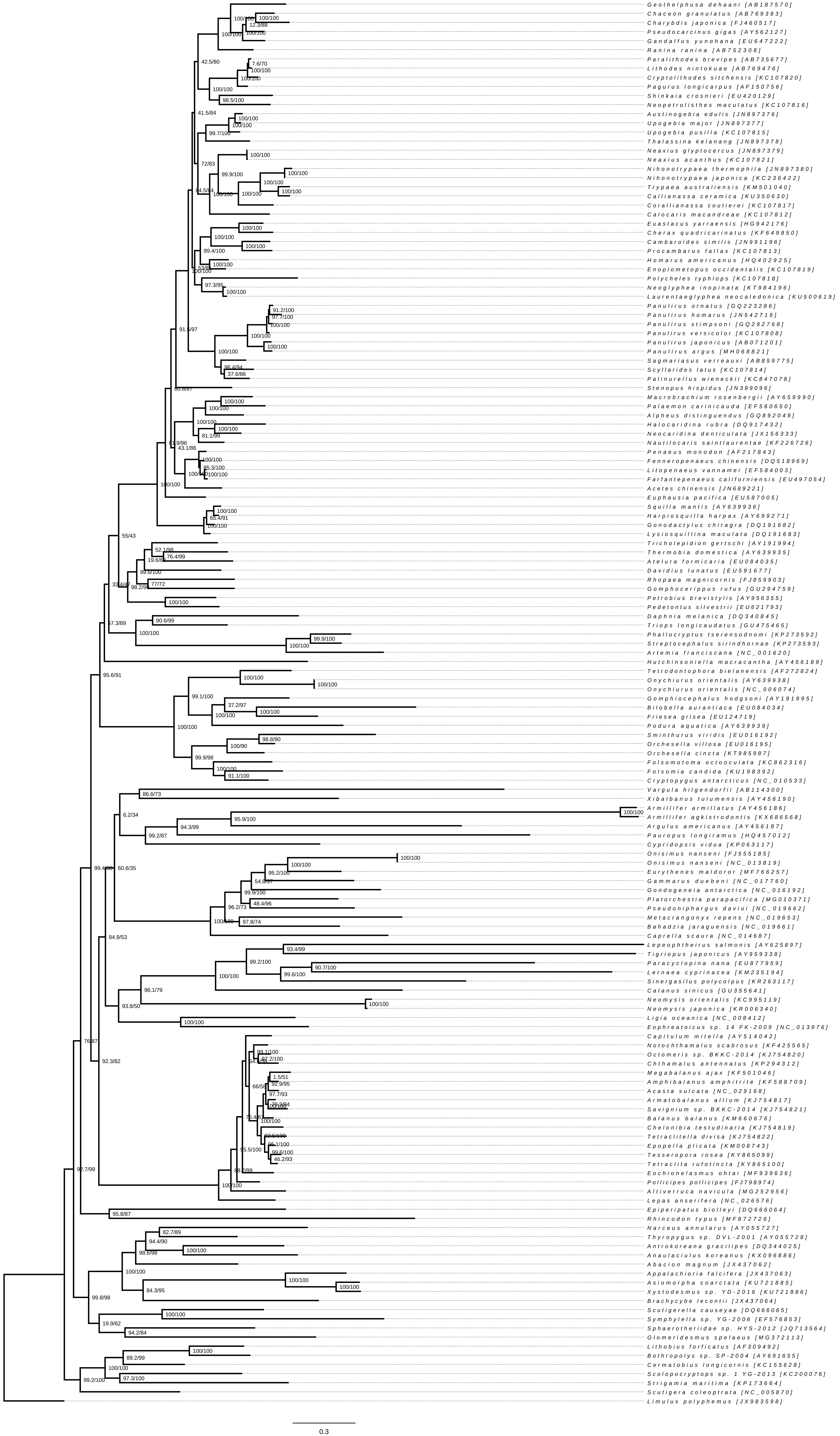
Geothelphusa dehaani [AB187570]
Chaceon granulatus [AB769383]
Charybdis japonica [FJ460517]
Pseudocarcinus gigas [AY562127]
Gandalfus yunohana [EU647222]
Ranina ranina [AB752308]
Paralithodes brevipes [AB735677]
Lithodes nintokuae [AB769476]
Cryptolithodes sitchensis [KC107820]
Pagurus longicarpus [AF150756]
Shinkaia crosnieri [EU420129]
Neopetrolisthes maculatus [KC107816]
Austinogebia edulis [JN897376]
Upogebia major [JN897377]
Upogebia pusilla [KC107815]
Thalassina kelanang [JN897378]
Neaxius glyptocercus [JN897379]
Neaxius acanthus [KC107821]
Nihonotrypaea thermophila [JN897380]
Nihonotrypaea japonica [KC236422]
Trypaea australiensis [KM501040]
Callianassa ceramica [KU350630]
Corallianassa coutierei [KC107817]
Calocaris macandreae [KC107812]
Euastacus yarraensis [HG942176]
Cherax quadricarinatus [KF649850]
Cambaroides similis [JN991196]
Procambarus fallax [KC107813]
Homarus americanus [HQ402925]
Enoplometopus occidentalis [KC107819]
Polycheles typhlops [KC107818]
Neoglyphea inopinata [KT984196]
Laurentaeglyphea neocaledonica [KU500619]
Panulirus ornatus [GQ223286]
Panulirus homarus [JN542716]
Panulirus stimpsoni [GQ292768]
Panulirus versicolor [KC107808]
Panulirus japonicus [AB071201]
Panulirus argus [MH068821]
Sagmariasus verreauxi [AB859775]
Scyllarides latus [KC107814]
Palinurellus wieneckii [KC847078]
Stenopus hispidus [JN399096]
Macrobrachium rosenbergii [AY659990]
Palaemon carinicauda [EF560650]
Alpheus distinguendus [GQ892049]
Halocaridina rubra [DQ917432]
Neocaridina denticulata [JX156333]
Nautilocaris saintlaurentae [KF226726]
Penaeus monodon [AF217843]
Fenneropenaeus chinensis [DQ518969]
Litopenaeus vannamei [EF584003]
Farfantepenaeus californiensis [EU497054]
Acetes chinensis [JN689221]
Euphausia pacifica [EU587005]
Squilla mantis [AY639936]
Harpiosquilla harpax [AY699271]
Gonodactylus chiragra [DQ191682]
Lysiosquillina maculata [DQ191683]
Tricholepidion gertschi [AY191994]
Thermobia domestica [AY639935]
Atelura formicaria [EU084035]
Davidius lunatus [EU591677]
Rhopaea magnicornis [FJ859903]
Gomphocerippus rufus [GU294759]
Petrobius brevistylis [AY956355]
Pedetontus silvestrii [EU621793]
Daphnia melanica [DQ340845]
Triops longicaudatus [GU475465]
Phallocryptus tserensodnomi [KP273592]
Streptocephalus sirindhornae [KP273593]
Artemia franciscana [NC_001620]
Hutchinsoniella macracantha [AY456189]
Tetrodontophora bielanensis [AF272824]
Onychiurus orientalis [AY639938]
Onychiurus orientalis [NC_006074]
Gomphiocephalus hodgsoni [AY191995]
Bilobella aurantiaca [EU084034]
Friesea grisea [EU124719]
Podura aquatica [AY639939]
Sminthurus viridis [EU016192]
Orchesella villosa [EU016195]
Orchesella cincta [KT985987]
Folsomotoma octooculata [KC862316]
Folsomia candida [KU198392]
Cryptopygus antarcticus [NC_010533]
Vargula hilgendorfii [AB114300]
Xibalbanus tulumensis [AY456190]
Armillifer armillatus [AY456186]
Armillifer agkistrodontis [KX686568]
Argulus americanus [AY456187]
Pauropus longiramus [HQ457012]
Cypridopsis vidua [KP063117]
Onisimus nanseni [FJ555185]
Onisimus nanseni [NC_013819]
Eurythenes maldoror [MF766257]
Gammarus duebeni [NC_017760]
Gondogeneia antarctica [NC_016192]
Platorchestia parapacifica [MG010371]
Pseudoniphargus daviui [NC_019662]
Metacrangonyx repens [NC_019653]
Bahadzia jaraguensis [NC_019661]
Caprella scaura [NC_014687]
Lepeophtheirus salmonis [AY625897]
Tigriopus japonicus [AY959338]
Paracyclopina nana [EU877959]
Lernaea cyprinacea [KM235194]
Sinergasilus polycolpus [KR263117]
Calanus sinicus [GU355641]
Neomysis orientalis [KC995119]
Neomysis japonica [KR006340]
Ligia oceanica [NC_008412]
Eophreatoicus sp. 14 FK-2009 [NC_013976]
Capitulum mitella [AY514042]
Notochthamalus scabrosus [KF425565]
Octomeris sp. BKKC-2014 [KJ754820]
Chthamalus antennatus [KP294312]
Megabalanus ajax [KF501046]
Amphibalanus amphitrite [KF588709]
Acasta sulcata [NC_029168]
Armatobalanus allium [KJ754817]
Savignium sp. BKKC-2014 [KJ754821]
Balanus balanus [KM660676]
Chelonibia testudinaria [KJ754819]
Tetraclitella divisa [KJ754822]
Epopella plicata [KM008743]
Tesseropora rosea [KY865099]
Tetraclita rufotincta [KY865100]
Eochionelasmus ohtai [MF939636]
Pollicipes pollicipes [FJ798974]
Altiverruca navicula [MG252956]
Lepas anserifera [NC_026576]
Epiperipatus biolleyi [DQ666064]
Rhincodon typus [MF872726]
Narceus annularus [AY055727]
Thyropygus sp. DVL-2001 [AY055728]
Antrokoreana gracilipes [DQ344025]
Anaulaciulus koreanus [KX096886]
Abacion magnum [JX437062]
Appalachioria falcifera [JX437063]
Asiomorpha coarctata [KU721885]
Xystodesmus sp. YD-2016 [KU721886]
Brachycybe lecontii [JX437064]
Scutigerella causeyae [DQ666065]
Symphylella sp. YG-2006 [EF576853]
Sphaerotheriidae sp. HYS-2012 [JQ713564]
Glomeridesmus spelaeus [MG372113]
Lithobius forficatus [AF309492]
Bothropolys sp. SP-2004 [AY691655]
Cermatobius longicornis [KC155628]
Scolopocryptops sp. 1 YG-2013 [KC200076]
Strigamia maritima [KP173664]
Scutigera coleoptrata [NC_005870]
Limulus polyphemus [JX983598]
0.3
